# OsZIP1 functions as a metal efflux transporter limiting excess zinc, copper and cadmium accumulation in rice

**DOI:** 10.1186/s12870-019-1899-3

**Published:** 2019-06-27

**Authors:** Xue Song Liu, Sheng Jun Feng, Bai Qing Zhang, Meng Qi Wang, Hong Wei Cao, Justice Kipkoir Rono, Xi Chen, Zhi Min Yang

**Affiliations:** 1 Department of Biochemistry and Molecular Biology, College of Life Science, Nanjing Agricultural University, Nanjing, 210095 China; 2 Zhejiang Provincial Key Laboratory of Bioremediation of Soil Contamination, Laboratory of Plant Molecular and Developmental Biology, Zhejiang Agriculture & Forestry University, Hangzhou, 311300 China

**Keywords:** OsZIP1, Rice, Endoplasmic reticulum, Metal efflux, Cadmium, DNA demethylation

## Abstract

**Background:**

Metal homeostasis is critical for plant growth, development and adaptation to environmental stresses and largely governed by a variety of metal transporters. The plant ZIP (*Z*n-regulated transporter, *I*ron-regulated transporter-like *P*rotein) family proteins belong to the integral membrane transporters responsible for uptake and allocation of essential and non-essential metals. However, whether the ZIP family members mediate metal efflux and its regulatory mechanism remains unknown.

**Results:**

In this report, we provided evidence that OsZIP1 is a metal-detoxified transporter through preventing excess Zn, Cu and Cd accumulation in rice. OsZIP1 is abundantly expressed in roots throughout the life span and sufficiently induced by excess Zn, Cu and Cd but not by Mn and Fe at transcriptional and translational levels. Expression of OsZIP-GFP fusion in rice protoplasts and tobacco leaves shows that OsZIP1 resides in the endoplasmic reticulum (ER) and plasma membrane (PM). The yeast (*Saccharomyces cerevisiae*) complementation test shows that expression of OsZIP1 reduced Zn accumulation. Transgenic rice overexpressing *OsZIP1* grew better under excess metal stress but accumulated less of the metals in plants. In contrast, both *oszip1* mutant and RNA interference (RNAi) lines accumulated more metal in roots and contributed to metal sensitive phenotypes. These results suggest OsZIP1 is able to function as a metal exporter in rice when Zn, Cu and Cd are excess in environment. We further identified the DNA methylation of histone H3K9me2 of *OsZIP1* and found that *OsZIP1* locus, whose transcribed regions imbed a 242 bp sequence, is demethylated, suggesting that epigenetic modification is likely associated with OsZIP1 function under Cd stress.

**Conclusion:**

OsZIP1 is a transporter that is required for detoxification of excess Zn, Cu and Cd in rice.

**Electronic supplementary material:**

The online version of this article (10.1186/s12870-019-1899-3) contains supplementary material, which is available to authorized users.

## Background

Metal homeostasis is a mechanism essential for plant growth, development and adaptation to diverse environmental stresses [1–3]. It is built up by metal uptake and transport across cells or within the cells, which largely relies on a variety of metal transporters [4, 5]. The plant ZIP (*Z*n-regulated transporter, *I*ron-regulated transporter-like *P*rotein) proteins belong to the metal transporter family involved in uptake and allocation of Zn, Cu, Cd, iron (Fe) or manganese (Mn) [1, 2, 6–8]. In Arabidopsis the ZIP family genes encode 14 protein members. AtIRT1 is a high affinity Fe uptake transporter [9–12]. AtIRT2 and AtIRT3 are responsible for Fe and Zn uptake in roots [13, 14]. AtZIP1 serves as a vacuolar transporter remobilizing Mn and Zn from vacuole to cytoplasm in root cells [12, 15]. AtZIP2 is located in the PM and may mediate Mn (and possibly Zn) uptake into root stele cells or Mn/Zn mobility in the stele to the xylem parenchyma [12]. While AtZIP3 is supposed to transport Zn and Fe from soil to plant [16], AtZIP4 is likely involved in Zn transport across tissues [2]. The rest of AtZIPs transport Mn, Zn and Fe [12]. The rice genome houses 18 ZIP members [17]. *OsIRT1* and *OsIRT2* are homologs primarily for Fe and Zn transport in roots [18, 19]. *OsZIP1* is thought of a Zn uptake transporter whose expression is induced under Zn deficiency [20–22], however, its biological functions in rice under metal stress are not fully understood. OsZIP2–5 and OsZIP8 are expressed in roots for Zn uptake or distribution [20, 21, 23–27]. OsZIP6 is induced in rice when Fe, Zn and Mn are deprived [28]. OsZIP7 is expressed in shoots under Zn deficiency [18] and in roots when Fe is limiting [26]. The functions of OsZIP9-OsZIP16 are less understood.

Cadmium is a non-essential and toxic metal to plants. Uptake of excess Cd jeopardizes crop production and food security [29]. Since no specific transporters for Cd are available, uptake of Cd into cells is shared by Fe, Zn and Mn transport systems [29]. To date, only a few studies are available on the transport of Cd out of plants. AtPDR8 is a PM transporter and can be induced by Cd exposure; mutantion of AtPDR8 leads to hypersensitivity to Cd, whereas AtPDR8 overexpression plants show Cd tolerance and accumulate less of Cd than wild-type [30]. Another example is rice Cal1 which can chelate Cd in cytosol and get it into the outer space [31]. Recently, the epigenetic modifications such as DNA methylation, histone modification and small interference RNAs have emerged as additional modulators for plant adaptation to biotic and abiotic stresses [32–38]. DNA methylation is a kind of post-modification predominantly found in cytosine residues (5-methyl cytosine, 5mC) of the dinucleotides of CG and to less extent, of CHG and CHH (where H is A, C or T). In plants, addition of a methyl group to the cytosine residue in the three contexts is mediated by a group of DNA methyltransferases such as MET1 (METHYLTRANSFERASE 1) mainly for CG, CMT3 (CHROMOMETHYLASES) for CHG, and DRM2 (DOMAINS REARRANGED METHYLTRANSFERASE 2) for CHH [39, 40]. Since DNA methylation/demethylation is a dynamic process, some demethylation enzymes such as ROS1 (REPRESSOR OF SILENCING), DME (DEMETER), DML2 (DEMETER-LIKE) and DML3 [41] are also involved in the process. While DNA methylation is the major epigenetic mechanism for regulating gene expression, the histone modifications at lysine or arginine are also engaged in the epigenetic process [42]. For example, the histone methylation at H3K9me2 is essential for DNA methylation particularly at the CHG catalyzed by CMT3 [43]. Studies demonstrate that Cd exposure can alter the genomic DNA methylation pattern in plants [37, 44, 45]. However, whether Cd-induced DNA methylation marks are able to mediate transcription of target genes and functional consequences is largely unknown.

Rice (*Oryza sativa* L.) is a model plant species for environmental research owning to its abundant germplasm resources. It is also one of the major crops. Many rice cultivars genotypes are gifted with special traits contributing to accumulating essential and nonessential metals or gain defense and tolerant traits [37, 46–48]. Understanding the regulatory mechanism for uptake, transport and accumulation of toxic metals like Cd is critical for developing strategies of hyper-accumulating or minimizing the toxic metals in crops growing on the metal-contaminated soils [29, 37, 49]. We previously identified several metal transporters including *OsZIP1* from transcriptome and methylome of Cd-exposed rice plants [37]. The underlying regulatory mechanisms for *OsZIP1* mediating Cd or other metals remain to be investigated. In this report, we provided the new evidence that *OsZIP1* is localized in both plasma membrane and endoplasmic reticulum. *OsZIP1* overexpression detoxified rice plants exposed to excess Zn, Cu and Cd by limiting accumulation of the metals in rice tissues. Furthermore, we precisely demonstrate that Cd exposure induced DNA and histone H3K9me2 demethylation in the transcribed region of *OsZIP1* which is likely associated with OsZIP1 function under Cd stress.

## Results

### Expression of *OsZIP1* is upregulated under excess Zn and Cu and Cd stress

The genomic sequence of *OsZIP1* (LOC Os01g74110) is 1909 bp in length, with a 1059 bp coding DNA sequence (CDS). The CDS was predicted to encode a 352-amino acid protein with eight transmembrane domains (TMDs) and a variable region between the 3th and 4th TMDs relevant to the metal binding and transport (Additional file 1: Figure S1).

**Fig. 1:**
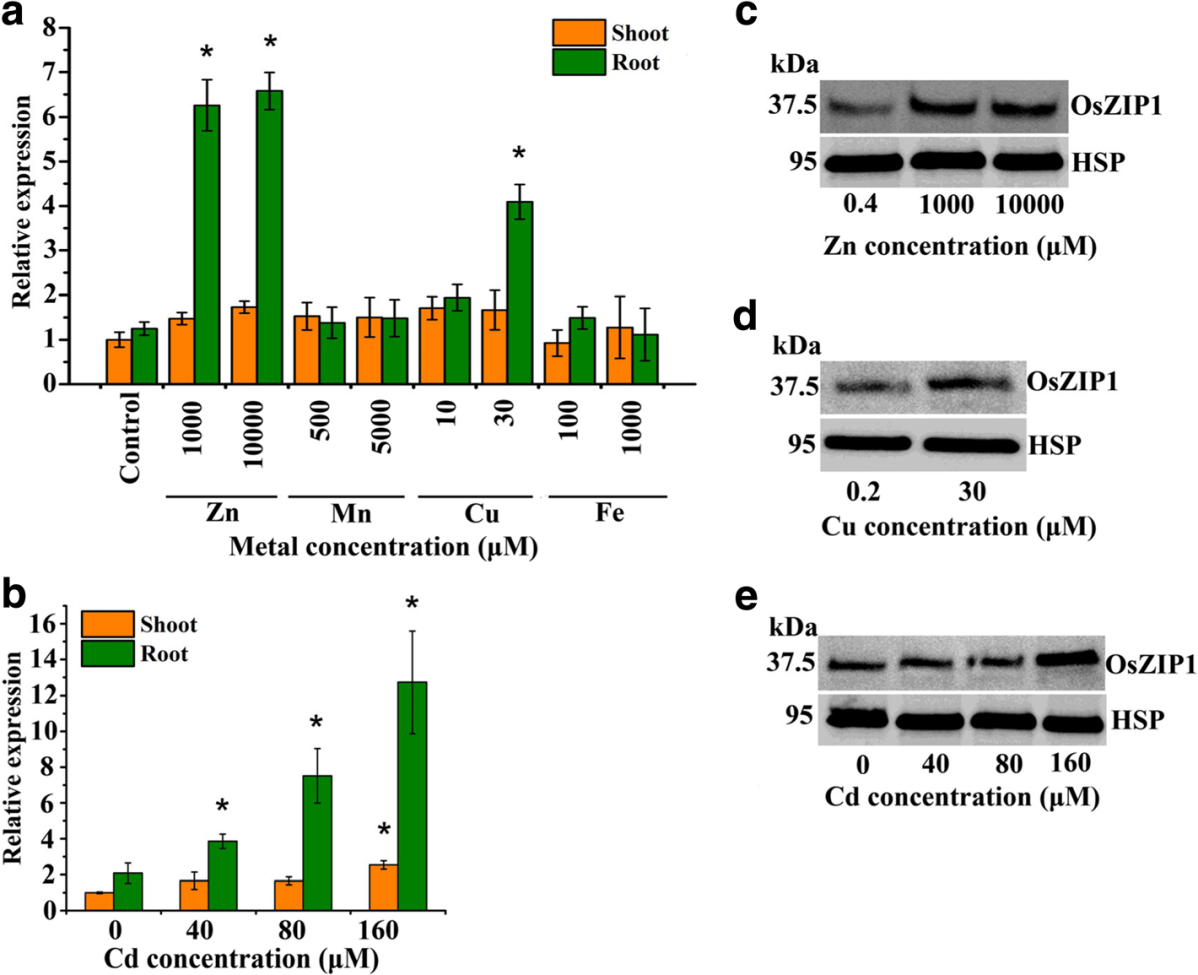
Analyses of OsZIP1 expression under metal stress conditions using qRT-PCR and Western blot. Two week-old rice plants grew in the nutrient solutions with 1000 and 10,000 μM Zn, 500 and 5000 μM Mn, 10 and 30 μM Cu, 100 and 1000 μM Fe and 0–160 μM Cd for 3 h. For Western blot analysis, seedlings were exposed to the metals at the indicated concentrations for 4 d. **a** Response of *OsZIP1* transcription to excess Zn, Mn, Cu and Fe. **b** Response of *OsZIP1* transcription to Cd. **c**-**e** Western blotting analysis of OsZIP1 expression in roots under excess Zn and Cu and Cd stress. Vertical bars represent standard deviation. Asterisks indicate that the mean values of three replicates are significantly different between the treatments and control (*p* < 0.05)

A qRT-PCR analysis showed that *OsZIP1* was transcriptionally expressed in rice throughout the life span (Additional file 2: Figure S2). In roots *OsZIP1* was abundantly expressed, but its expression was weak in shoots. We sought to explore the challenge of metal excess to *OsZIP1* expression. Two week-old rice plants were exposed to the excess metals Zn, Cu, Fe, Mn and Cd, and the transcripts of *OsZIP1* were measured by qRT-PCR. A high level (> 6 fold) of *OsZIP1* transcripts was detected in roots exposed to 1–10 mM Zn over the control (Fig. 1a). Treatment with 30 μM Cu moderately affected expression of *OsZIP1*, with 4 fold over the control (Fig. 1a). There was no effect of excess Mn and Fe on *OsZIP1* transcripts. Transcripts of *OsZIP1* in roots were increased under 40–160 μM Cd exposure (Fig. 1b), which is similar to the previous study [37]. The *pOsZIP1::GUS* vector was constructed and transformed into tobacco leaves by *Agrobacterium tumefaciens* transformation. The *GUS* transcripts under Cd stress were increased more 2.5 fold than those under the control (Additional file 3: Figure S3). Expression of *OsZIP1* in roots induced by excess Zn, Cu and Cd was confirmed by Western blot analysis (Fig. 1c-e).

### OsZIP1 resides in the PM and ER

**Fig. 2:**
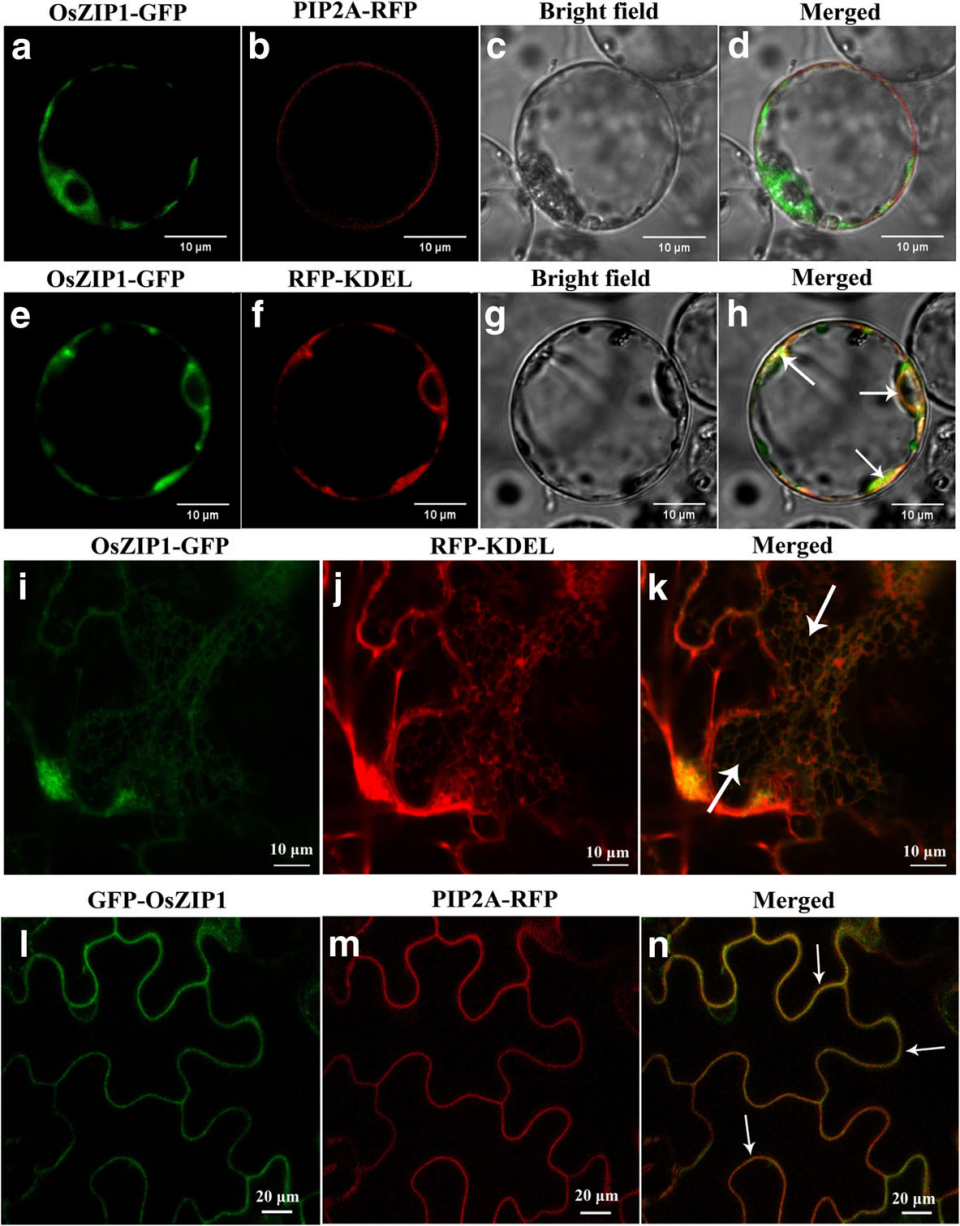
Subcellular localization of OsZIP1-GFP fusion proteins with plasma membrane (PM) and endoplasmic reticulum (ER) markers in rice mesophyll protoplast (**a-h**) and tobacco leaf epidermal cells (**i-k**) detected by Confocal images under normal metal supply. **a** OsZIP1-GFP. **b** PM marker PIP2A-RFP. **c** Bright field. **d** Merged images of a and b. **e** OsZIP1-GFP. **f** ER marker RFP-KDEL. **g** Bright field. **h** Merged images of e and f. **i** OsZIP1-GFP. **j** ER marker RFP-KDEL. **k** Merged images of i and j. **l** OsZIP1-GFP. **m** PM marker PIP2A-RFP. **n** Merged images of l and m. Arrows indicate the part of the ER or PM areas where OsZIP1-GFP fusion images were overlapped with the corresponding markers

OsZIP1 is expressed in the vascular bundles and epidermal cells in rice roots, and is proposed to be a Zn uptake transporter [20]. We therefore constructed a recombinant plasmid connecting the green fluorescent protein (GFP) to *OsZIP1*, which was expressed in the rice mesophyll protoplasts. Confocal microscopy analysis revealed overlapped PM biomarker fluorescence (PIP2A-RFP, red) and OsZIP1-GFP fluorescence (green) signals in the rice mesophyll protoplasts (Fig. 2 a-d). We then tested several other specific biomarkers and found that only ER marker (RFP-KDEL, red) was probed, with strong signals overlapped with the fusion proteins (Fig. 2 e-h). To confirm the observation, the OsZIP1-GFP fusion was transformed and expressed in tobacco leaf epidermal cells [50]. Using the same ER marker, clear signals of co-localization of the fusion proteins and marker around the ER area were visualized (Fig. 2 i-k). Moreover, strong signals of GFP only and the merged images of GFP and PM-marker were detected (Fig. 2 l-n), suggesting that OsZIP1 is also localized to plasma membrane.

### Expressing *OsZIP1* in yeast reduced Zn accumulation in cells

The wild-type and mutants of yeast (*Saccharomyces cerevisiae*) were used to identify the role of *OsZIP1* in mediating metal resistance and accumulation in the cells. The empty vector pYES2 and the vector carrying *OsZIP1* were transformed into the *zrc1* mutants. *OsZIP1-*transformed *zrc1* cells exposed to 3 and 6 mM Zn showed more resistance to excess Zn than the empty vector cells (Additional file 4: Figure S4). Analyses by ICP-AES revealed that the *OsZIP1*-expressing yeast cells accumulated significantly lower levels of Zn than its control cell (Additional file 4: Figure S4).

### OsZIP1 knockdown repressed and OX lines promoted the rice growth under excess Zn, Cu and Cd stress

**Fig. 3:**
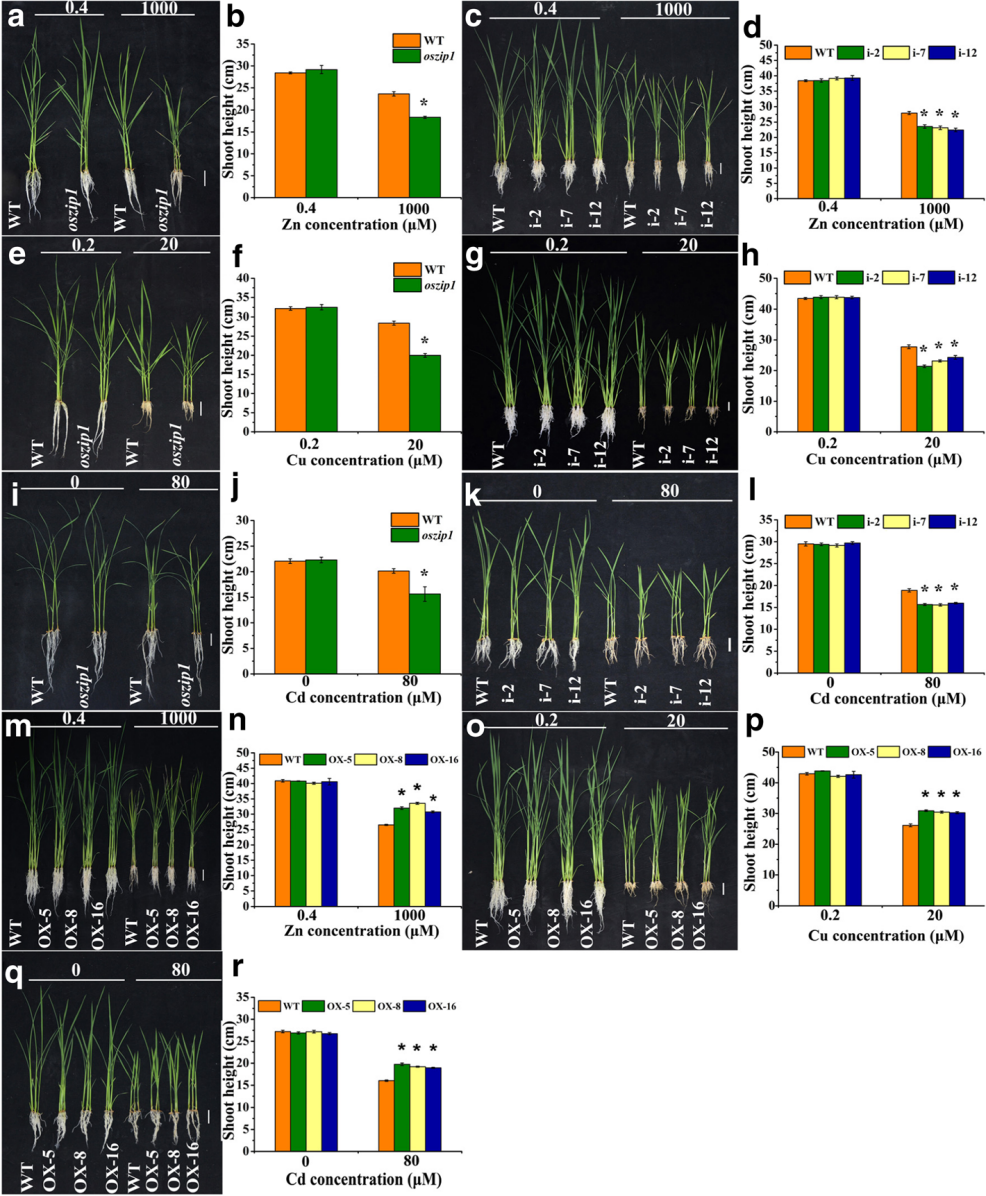
Growth response to excessive Zn, Cu and Cd stress in rice wild-type (WT), *oszip1* mutant, RNAi lines (i) and *OsZIP1*-transgenic lines (OX). Two weeks-old young rice plants were grown in the nutrient solution supplemented with 0.4 (normal) and 1000 (high) μM Zn or 0.2 (normal) and 20 (high) μM Cu for 10 days or with 0 and 80 μM Cd for 4 d. **a-d**, **m**, **n** Phenotypes and shoot length of WT, *oszip1*, RNAi and OX plants exposed to Zn excess. **e-h**, **o**, **p** Phenotypes and shoot length of WT, *oszip1*, RNAi and OX plants exposed to Cu excess. **i-l**, **q**, **r** Phenotypes and shoot length of WT, *oszip1*, RNAi and OX plants exposed to Cd. Vertical bars represent standard deviation. Asterisks indicate that the mean values are significantly different between the *oszip1* mutant/RNAi lines/OX lines and wild-type (*p* < 0.05)

**Fig. 4:**
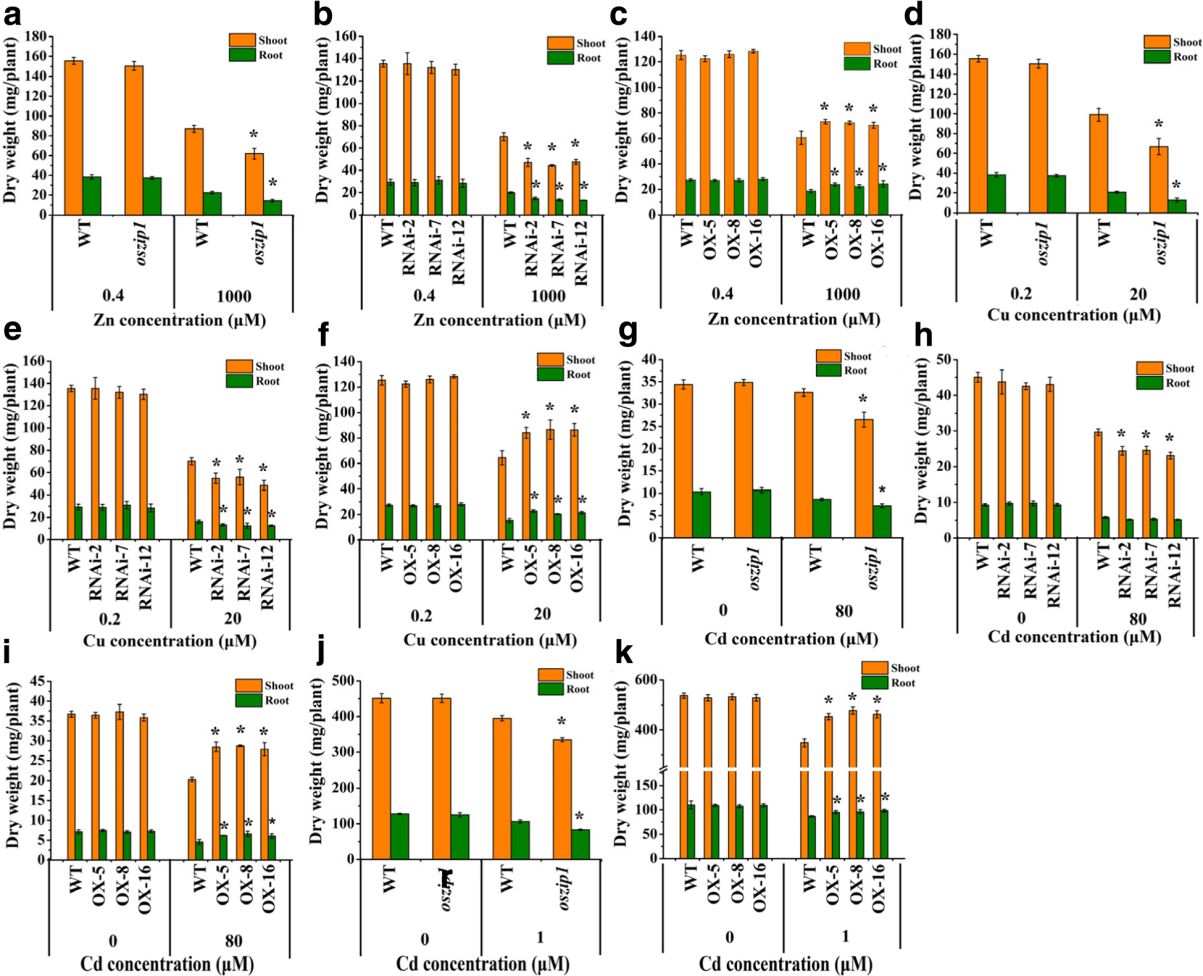
Growth response to excess Zn, Cu and Cd stress in wild-type (WT), *oszip1* mutant, RNAi and OX lines. Two weeks-old young rice plants were grown in the nutrient solution supplemented with 0.4 (normal) and 1000 (high) μM Zn or 0.2 (normal) and 20 (high) μM Cu for 10 days or with 0 and 80 μM Cd for 4 d or 1 μM Cd for 30 d. **a-c** Dry weight of WT, *oszip1*, RNAi and OX plants exposed to Zn excess. **d-f** Dry weight of WT, *oszip1*, RNAi and OX plants exposed to Cu excess. **g-k** Dry weight of WT, *oszip1*, RNAi and OX plants exposed to Cd. Vertical bars represent standard deviation. Asterisks indicate that the mean values are significantly different between the transgenic plants/mutants and wild-type (*p* < 0.05)

A T-DNA insertion mutant *oszip1* was ordered from the rice database [51]. PCR analysis showed a single insertion in the intron (Additional file 5: Figure S5a). The *OsZIP1* transcripts in the *oszip1* line were reduced to 10.0% of its wild-type and the protein level was also drastically lowered (Additional file 5: Figure S5 b-d). Additionally, a set of independent knockdown lines of *OsZIP1* were generated using RNA interference approach. Transcript analysis showed that the RNAi lines contained only 20.1–30.2% transcripts of the wild-type (Additional file 5: Figure S5 e). We experimented with the knockdown lines exposed to the high level of metals for a short time and the low level of metals for a long time. Under control condition, no differences in growth response between *oszip1* or RNAi lines and wild-type were observed; however, when under 1000 μM Zn, 20 μM Cu or 80 μM Cd, both *oszip1* and RNAi lines displayed the reduced shoot growth and dry weight compared to the wild-type (Fig. 3 a-l; Fig. 4 a,b, d, e, g, h). A similar response was found with the long-term study (Fig. 4 j).

We further generated *OsZIP1*-overexpressing lines (OXs) under the control of cauliflower mosaic virus 35S promoter. The OX lines used in this study showed 25.1 to 41.5-fold higher transcripts of *OsZIP1* and higher protein levels than the wild-type (Additional file 5: Figure S5f, g). There were no growth differences between the OX lines and wild-type plants grown in the non-Cd medium (Fig. 3 m-r). When exposed to 1000 μM Zn, 20 μM Cu or 80 μM Cd, the shoot of OX lines grew longer, with 1.3–1.5 fold for Zn, 1.2–1.3 fold for Cu, and 1.2–1.3 fold for Cd over the wild-type (Fig. 6 m-r). The enhanced dry biomass was obtained in the OX lines (Fig. 4 c, f, i, k).

### OsZIP1 knockdown and OX lines depicted a contrasting metal accumulation under excess metal stress

**Fig. 5:**
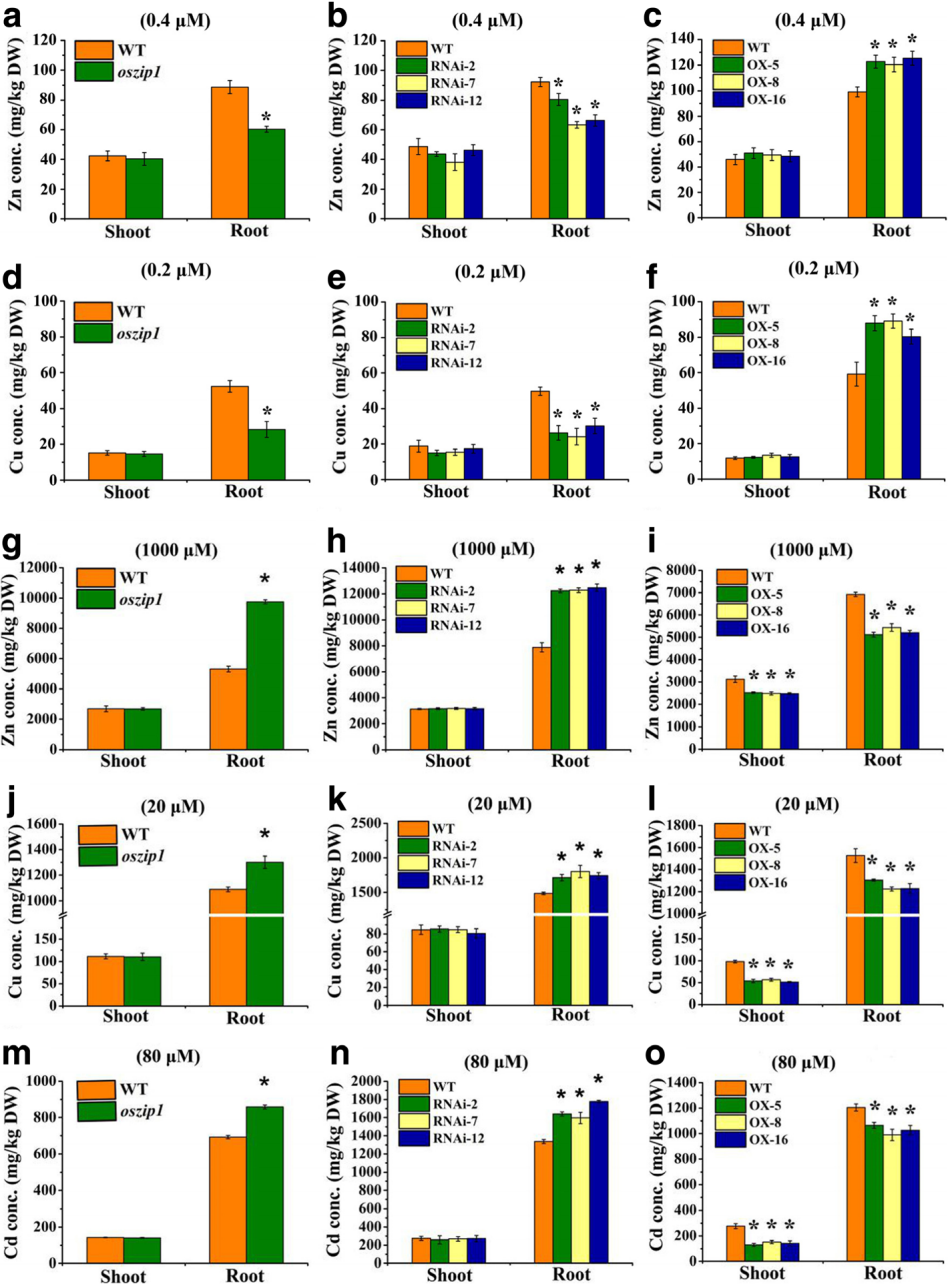
Zn, Cu and Cd concentrations in shoots and roots of WT, *oszip1* mutant, RNAi lines and OX lines under excess metal stress. Two week-old rice seedlings were grown in nutrient solutions supplemented with 1000 μM Zn or 20 μM Cu for 10 d or supplemented with 80 μM Cd for 4 d. 0.4 μM Zn and 0.2 μM Cu were used as the controls. Zn **(a-c**) and Cu (**d-f**) concentrations in WT and *oszip1* mutants, RNAi lines and OXs lines under normal conditions. Zn **(g-i**) and Cu (j**-l**) concentrations in WT, *oszip1* mutant, RNAi lines and OX lines under excess Zn and Cu stress. **m-o** Cd concentrations in WT, *oszip1* mutant, RNAi lines and OX lines exposed to 80 μM. Vertical bars represent standard deviation. Asterisks indicate that the mean values are significantly different between the *oszip1* mutant/ RNAi lines/OX lines and wild-type (*p* < 0.05)

Under normal conditions (0.4 μM Zn and 0.2 μM Cu), the *oszip1/*RNAi, OX and wild-type plants had the similar Zn and Cu concentrations in their shoots. In roots, the *oszip1/*RNAi lines had a lower concentration of Zn and Cu, while the OX lines had a higher level of the metals compared to the wild-type (Fig. 5 a-f). Examination of metal concentrations in plants exposed to the excess levels (1000 μM Zn, 20 μM Cu and 80 μM Cd) shows that both *oszip1* and RNAi lines accumulated more Zn, Cu and Cd in their roots than the wild-type, whereas no differences in metal concentrations were observed in the shoots (Fig. 5 g, h, j, k, m, n). Under the same conditions, the OX lines accumulated less Zn, Cu and Cd in roots and shoots than the wild-type (Fig. 5 i, l, o). The OsZIP1-mediated metal accumulation is similar to the previous report on a metal efflux transporter OsHMA9, as mutation of OsHMA9 led to increased accumulation of more Zn, Cu and Cd in roots [52].

**Fig. 6:**
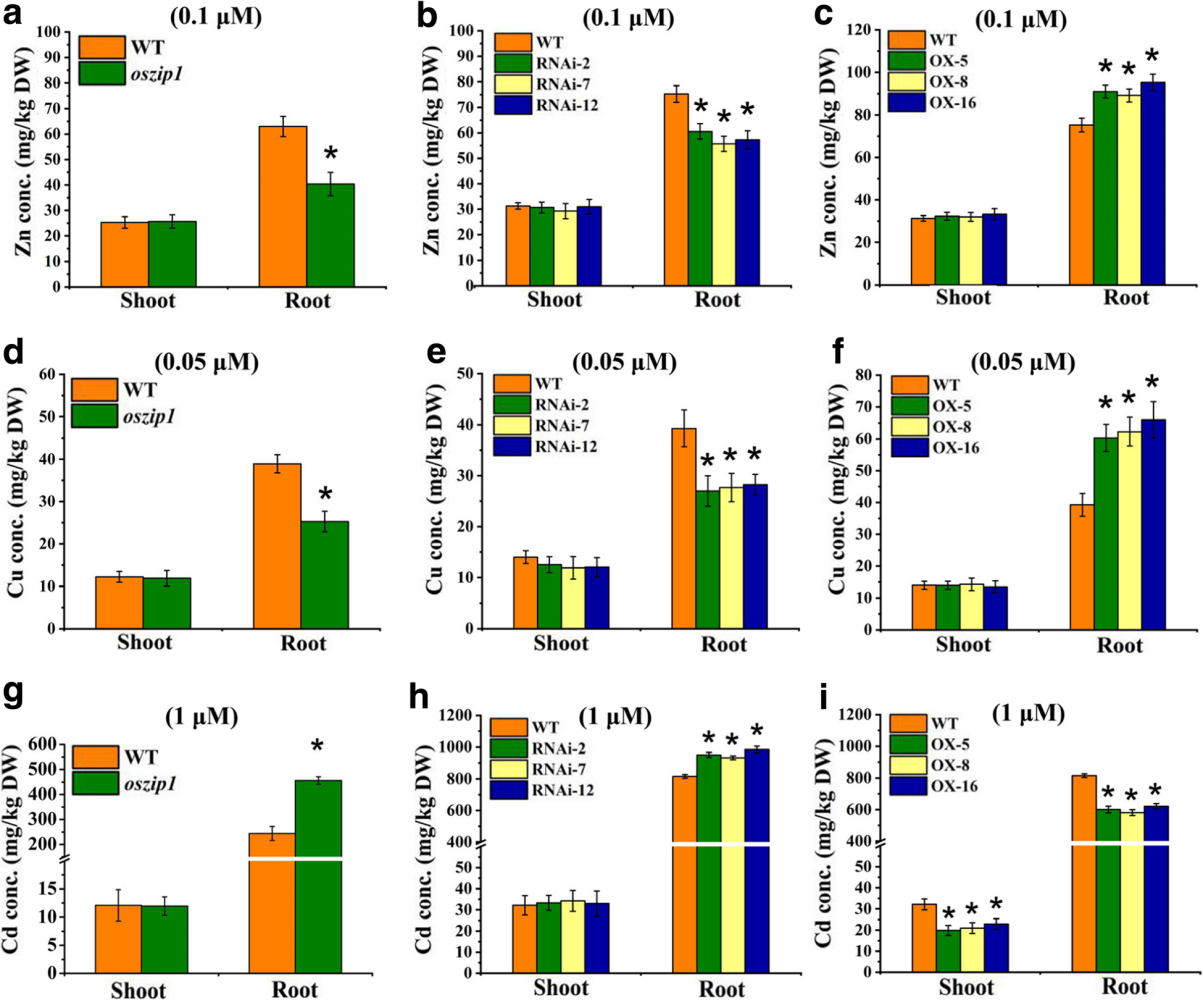
Zn, Cu and Cd concentrations in shoots and roots of WT, oszip1 mutant, RNAi lines and OX lines exposed to limiting Zn and Cu or lower concentration of Cd. Two day-old young plants were grown in the nutrient solutions with 0.1 µM Zn (**a**-**c**), 0.2 µM Cu (**d**-**f**) or supplemented with 1 µM Cd (**g**-**i**) for two weeks. Vertical bars represent standard deviation. Asterisks indicate that the mean values are significantly different between the oszip1 mutant/ RNAi lines/OX lines and wild-type (*p*<0.05)

We identified the role of OsZIP1 in mediating accumulation of metals under low supply conditions. Treatments with the lower levels of Zn (0.1 μM) and Cu (0.05 μM) led to lower accumulation of Zn and Cu in the roots of *oszip1* and RNAi lines but higher accumulation in the OX lines, compared to the normal condition (Fig. 6 a-f). Analyzing Cd concentration in rice exposed to the lower level of Cd showed a higher concentration of Cd in the *oszip1* and RNA1 roots but no difference in shoots (Fig. 6 g-i). These results suggested that knockdown of *OsZIP1* led to lower accumulation of Zn and Cu but higher accumulation Cd in roots, whereas *OsZIP1* overexpression led to a contrasting metal accumulation.

### Cd stress reduced DNA methylation in the transcribed region of *OsZIP1*

**Fig. 7:**
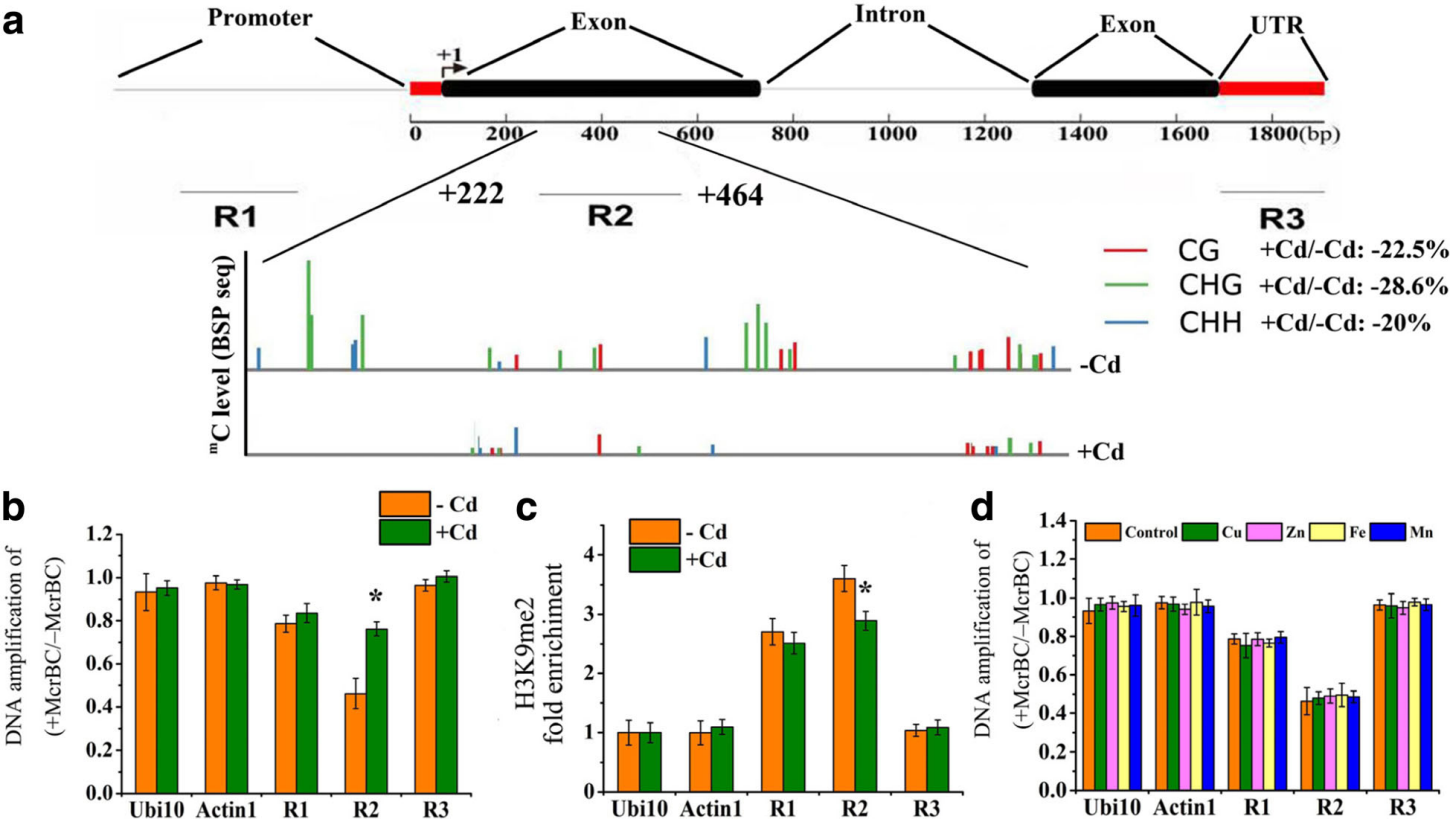
Identification of DNA and histone demethylation of *OsZIP1* in rice exposed to excess Zn, Cu, Mn, Fe and Cd. **a** Diagram of the *OsZIP1* genomic region with CG, CHG and CHH demethylation pattern in R2 with Cd (+Cd) and without Cd (−Cd) exposure. **b** DNA methylation status At R1-R3 of *OsZIP1* with -Cd and + Cd was determined by McrBC-qPCR assays (Note: a lower value of DNA amplification of (+McrBC/−McrBC) indicates the higher methylation). **c**: Histone methylation state was detected using an anti-H3K9me2 ChIP-qPCR assay at R1-R3 of the genomic regions of *OsZIP1* under –Cd and + Cd conditions. **d** DNA methylation status at R1-R3 of *OsZIP1* with the normal and excessive Zn, Cu, Mn and Fe supply. Two weeks-old young rice plants were grown in the nutrient solution supplemented with 0.4 (normal) and 1000 (high) μM Zn, 0.2 (normal) and 20 (high) μM Cu, 0.5 (normal) and 500 (high) μM Mn, and 10 (normal) and 1000 (high) μM Fe, or with 0 and 80 μM Cd for 4 d. The rice Ubi2 and Actin1 were tested in parallel as negative controls. Anti-H3 was used as an internal reference in the ChIP-qPCR assay. Vertical bars represent standard deviation. Asterisks indicate that the mean values of three replicates are significantly different between the metal treatments and control (*p* < 0.05)

Our previous studies show that Cd exposure is able to induce DNA demethylation of *OsZIP1*, however, the detailed epigenetic processes remain elusive. To clarify it, three regions including the promoter (R1), intragenic (gene body) (R2) and intergenic (downstream) (R3) sequences of *OsZIP1* were profiled (Fig. 7a). Using the BS-seq datasets [37], we found a significant demethylation region (+ 222~ + 464) within R2 under Cd stress (Fig. 7a), where the total cytosine methylation was reduced by 24.4% compared to the control (−Cd treatment); among the three contexts, CHG, CG and CHH methylation was reduced by 28.6, 22.5 and 20.0%, respectively. The Cd-induced body demethylation was confirmed by PCR-based DNA methylation assay (Fig. 7b). A long-term experiment with the rice plants exposed to 1 μM Cd for 30 days was performed, but the body demethylation at R2 of *OsZIP1* was not detected under the condition (Additional file 6: Figure S6). Similarly, the transcripts of *OsZIP1* did not increase in rice exposed to the low dose of Cd (Additional file 7: Figure S7).

H3K9me2 occurs around the repressed euchromatic regions and plays an important role in DNA methylation-mediated gene silencing [43, 53]. The rice *SDG714* encodes an H3K9me2-specific histone methyltransferase and coordinates CMT3 for DNA methylation mainly at the CHG sequence context [53–55]. By analyzing chromatin immunoprecipitation (ChIP) using a specific H3K9me2 antibody, we found the reduced H3K9me2 marks in the R2 transcribed regions of *OsZIP1* under Cd stress (Fig. 7c). The DNA methylation and H3K9me2 pattern was also investigated under the Zn and Cu stress condition, however, no change at the specific site was found (Fig. 7d). Likewise, both Fe and Mn excess failed to change the DNA methylation of *OsZIP1* (Fig. 7d).

**Fig. 8:**
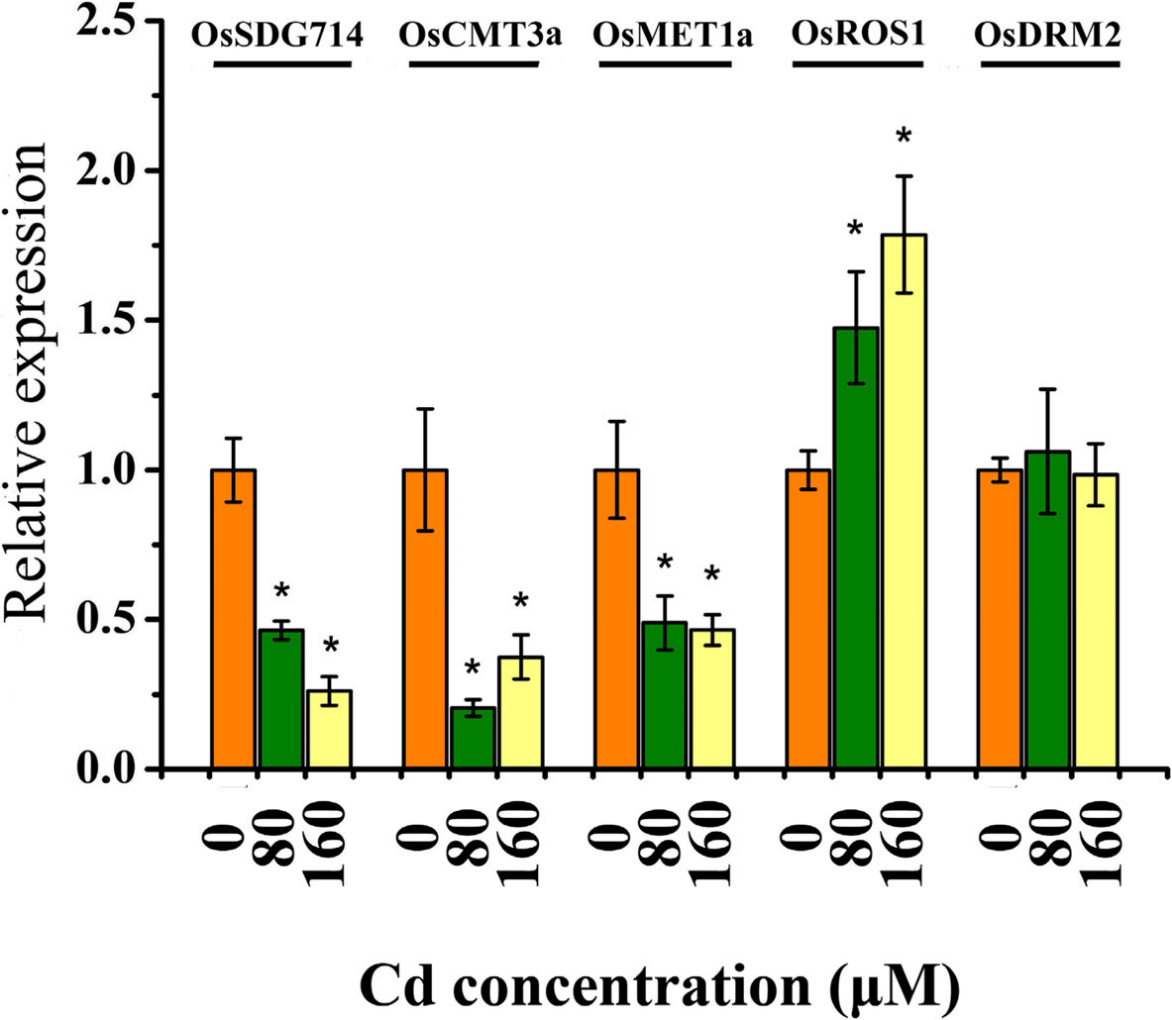
Effect of Cd on the transcripts of DNA methylation modifier genes. Two week-old young rice plants were grown in the nutrient solution supplemented with 0, 80 and 160 μM Cd for 4 d. Vertical bars represent standard deviation. Asterisks indicate that the mean values of three replicates are significantly different between the treatment and control (*p* < 0.05)

Since *OsZIP1* methylation pattern was affected by Cd, it is essential to investigate whether Cd stress could affect the expression of DNA methylation-related protein genes. To address the question, qRT-PCR was used to test the transcriptional response of several genes to Cd stress. The *OsCMT3a* expression was repressed in rice under Cd stress (Fig. 8). The reduced transcripts of *OsSDG714* and *OsMET1* were also found in rice under Cd stress (Fig. 8). In contrast, expression of *OsROS1* was slightly induced by Cd stress. Expression of *OsDRM2* had no response to Cd stress. We further examined the response of these genes in expose to the low Cd and found no significant changes (Additional file 8: Figure S8). Finally, transcripts of the genes under excess Zn, Cu, Fe and Mn were examined and none of them were significantly changed (Additional file 9: Figure S9).

### Mutation of *OsSDG714* led to increase in *OsZIP1* transcription

**Fig. 9:**
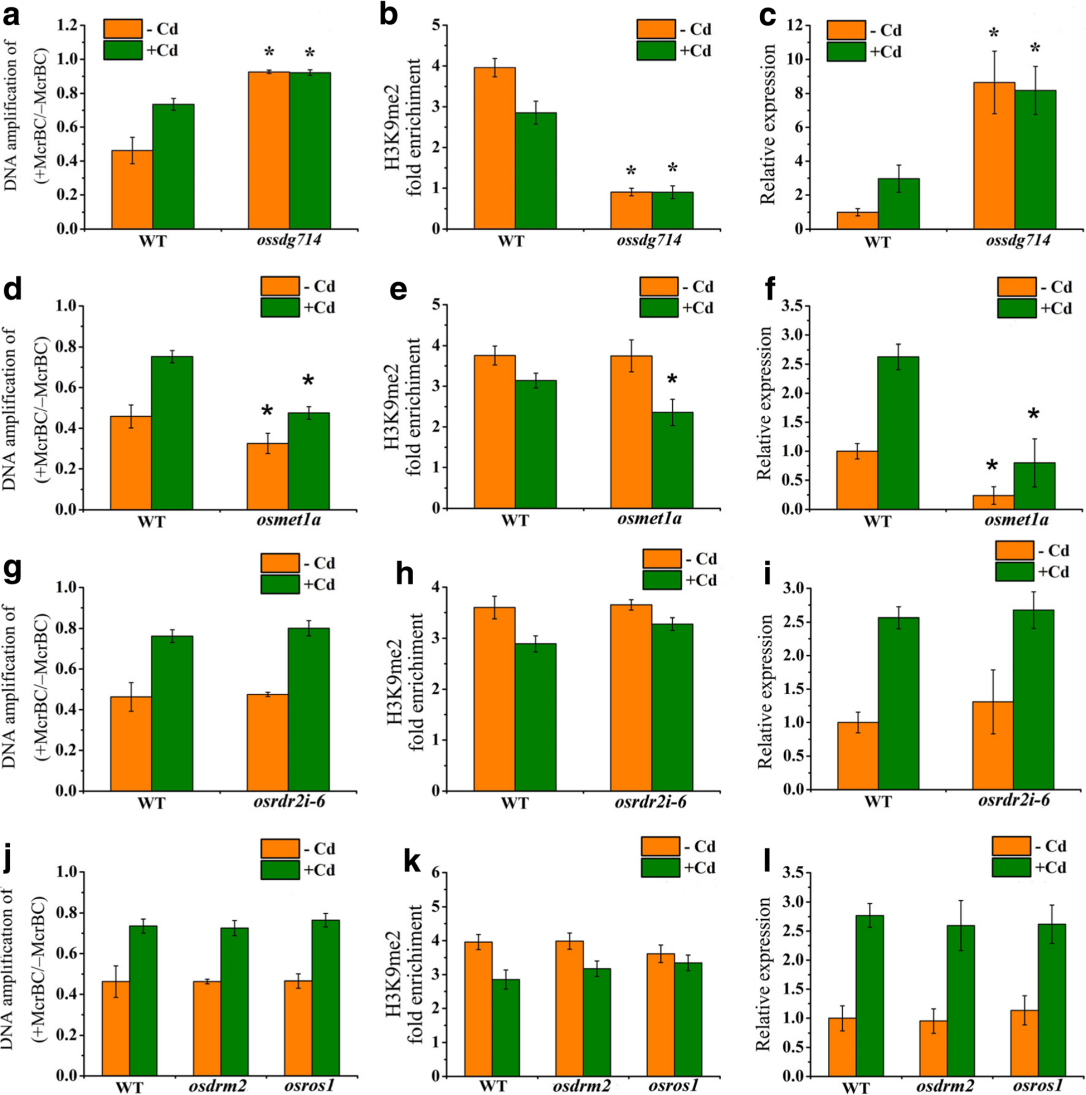
Identification of DNA and histone demethylation and expression of *OsZIP1* in the wild-type (WT) and epigenetic modified mutant plants exposed to Cd. DNA methylation (**a**, **d, g** and **j**), H3K9me2 mark (**b**, **e, h** and **K**) and transcript levels (**c**, **f, i** and **l**) of *OsZIP1* in *ossdg714*, *osmet1*, *osrdr2i-6*, *osdrum2* and *osros1* mutants and their wild-types with or without Cd exposure. Two weeks-old young rice plants were grown in the nutrient solution supplemented with 0 and 80 μM Cd for 4 d. The rice *Actin1* was used as a negative control. Anti-H3 was used as an internal reference in the ChIP-qPCR assay. Vertical bars represent standard deviation. Asterisks indicate that the mean values of three replicates are significantly different between the wild-type and mutants (*p* < 0.05)

We assessed the DNA methylation status and *OsZIP1* transcripts using a set of mutants defective in activities of DNA methylation/demethylation, histone modification and small RNA generation. Identification of T-DNA insertion of the mutants was previously described [37]. Since *oscmt3a* was unavailable and OsSDG714 is necessary for CHG methylation, the *ossdg714* mutant was used in the study. Mutation of *SDG714* caused massive loss of total H3K9me2 in *OsZIP1* under -Cd and + Cd conditions (Fig. 9a) and consequently, the CHG methylation of *OsZIP1* in *ossdg714* was significantly lost (Fig. 9b). Meanwhile, the *OsZIP1* transcripts were drastically increased (Fig. 9c), suggesting that loss of H3K9me2 and CHG methylation in *ossdg714* may associate the increased *OsZIP1* transcripts. We checked on the DNA methylation and H3K9me2 levels of *OsZIP1* in the rest of the mutants. The DNA methylation of *OsZIP1* in *osmet1* was increased (Fig. 9d). In the absence of Cd, there was no change in H3K9me2 marks between *osmet1* and wild-type plants; however, the H3K9me2 level in *osmet1* was significantly lower than those of wild-type under Cd stress (Fig. 9e). According to DNA methylation status, the transcript levels of *OsZIP1* in *osmet1* were significantly lower than those in wild-type (Fig. 9f). There was no change in DNA methylation and H3K9me2 marks as well as transcripts of *OsZIP1* in *osdrm2–2*, *osros1*, and *osrdr2i-6* (Fig. 9g-l).

### DNA methylation inhibitor-mediated detoxification of Cd-exposed rice depends on *OsZIP1*

**Fig. 10:**
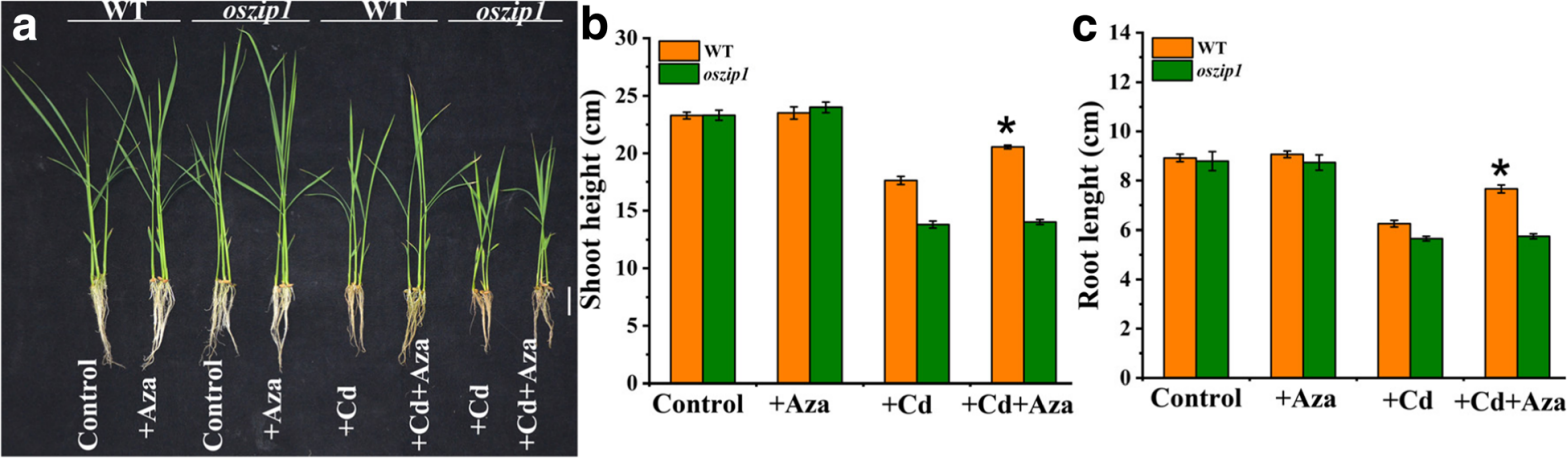
Effects of DNA methyltransferase inhibitor 5-aza-2-deoxycytidine (Aza) on the growth of *oszip1* mutants and wild-type rice plants exposed to Cd. Seven day-old young rice plants were treated with Cd (80 μM) and/or Aza (20 μM) for 4 d. Thereafter, the phenotype of rice seedlings were photographed (Scale bar = 2 cm). **a** Phenotype of rice plants. **b** Shoot length of rice plants. **c** Root length. Vertical bars represent standard deviation of the mean of three biological replicates. Asterisks indicate that the mean values are significantly different between the treatments (*p* < 0.05)

To verify that the *OsZIP1* demethylation was able to alter the phenotype of rice growth under Cd stress, we performed an additional experiment with a global DNA methylation inhibitor, azacitidine (Aza) [56]. Young rice plants were exposed to 80 μM Cd and 20 μM Aza for 4 d, and the growth response was assessed by measuring the shoot and root elongation. As shown in Fig. 10, Aza provision elongated the shoots and roots of wild-type under Cd stress, whereas no change in shoot or root growth of *oszip1* mutants was observed between the Cd and Cd + Aza treatments, indicating that Aza failed to relieve Cd-induced inhibition of shoot and root growth due to the mutation of *OsZIP1* and suggesting that Aza-mediated detoxification in Cd-exposed plants depends on *OsZIP1*.

## Discussion

The toxic heavy metal Cd is a non-essential element for plant growth and development, therefore, it must be under the tight control; likewise, even for essential metals such as Zn, Mn or Cu, the concentrations must be also restricted to a narrow range [29]. To prevent toxicity of excess metals, plants develop elaborate mechanisms for governing metal uptake, translocation and homeostasis. One of them is concerning the efflux transport systems. To date, several efflux transporters have been functionally characterized in rice [31, 52]. However, compared to those from Arabidopsis, the rice metal exporters are less reported.

OsZIP1 has long been considered as a metal uptake transporter for Zn and Cd in rice [15, 17, 21, 22]. Evidence to support the view relies on the increased transcripts of *OsZIP1* under Zn deficiency and the transport activity for Zn in a *OsZIP1*-transformed yeast (ZHY3) [15, 17, 21, 22]. This study presented several lines of new evidence that both *OsZIP1* transcripts and proteins can be substaintially incrased under excess Zn, Cu and Cd stress. Yeast complementation test supported the activity of OsZIP1 for the metal export and detoxification. In an accordace with it, OsZIP1 overexpression reduced concentrations of Zn, Cu and Cd in rice. As a consequence, the growth status was improved under metal stress. These results support the notion that OsZIP1 would be a transporter responsible for metal exfllux in rice. The phenotype of OsZIP1 resembles many metal efflux transporters such as OsHMA9 [52]; TcHMA4 [57]; AtHMA4 [58]; AtPDR8 [30]; CAL1 [31] and AtPCR2 [59].

Since most of the metal exporters localize to the PM, we addressed the question by examining OsZIP1 fusion proteins in both rice protoplasts and tobacco leaves. The fluorescence signals of OsZIP1-GFP occurred in both PM and ER. The dual localization suggests that OsZIP1 may play roles under changing environmental condition. Our data corroborate a model that has been recently illustrated from in Caryophyllaceae (*Silene vulgaris*) [60]. SvHMA5II is a Cu-tolerant transporter isolated from *S. vulgaris* and resides in the ER and upon Cu exposure, it re-localizes to the PM where the excess Cu is supposed to be fluxed out of cells [60]. Meanwhile, a study with yeast (*Saccharomyces cerevisiae*) provided a similar a model for extrusion of Pca1 (a P-type ATPase metal transporter) from the ER to PM, where it functions in the efflux of Cd across the PM [61]. Pca1 is a short-lived and primarily ER-localized protein that is targeted by a degron for ubiquitination and proteasomal degradation before approaching cell surface. Cd can upregulate Pca1 rapidly in the ER and lead to the degron bound to Pca1, which prevents Pca1 from being degraded in the so called endoplasmic reticulum-related degradation system [61]. Whether there is a similar metal exclusion mechanism in higher plants will be an interesting topic of research.

DNA methylation is an important epigenetic mechanism for regulating many plant biological processes [32, 35, 36, 38, 62]. Whether the epigenetic mechanism functionally participates in plant response to heavy metal stress is largely unknown. This study precisely characterized the *OsZIP1* methylation regions and found that the gene body is the region most demethylated under Cd stress. In euchromatin, genes with methylated regions in their promoters are usually those inducible, tissue-specific developmental-regulator or environmental stress response genes [63–65], whereas genes densely methylated in their transcribed regions are those actively expressed [66–71]. The gene body methylation has been thought to be conserved and to slowly evolve [66, 67, 71, 72]. However, compared to the promoter-methylated genes, the biological significance of the body-methylated genes relevant to the evolution, functionality and cytosine methylation pattern is poorly understood [63, 71]. Given that the body methylation preferentially occurs in the constitutively expressed genes [66, 67, 73] and is associated with active transcription [63, 70, 71], most of them are proposed to play pivotal roles in biological functions [71]. On the other hand, the body methylation genes tend to be weakly expressed and even be transcriptionally repressed [72, 74]. Our study is partially consistent with the view that the densely methylated context is associated with the low level of *OsZIP1* expression and, upon Cd exposure the methylation was reduced. The detailed mechanism for the dynamic methylation and demethylation change under Cd stress remains elusive. By identifying a suit of DNA methylation-defective mutants, only in *ossdg714* was found the methylation in the transcribed region of *OsZIP1* to be lower and the *OsZIP1* transcripts to be higher (Figs. 8, 9), suggesting that the reduced H3K9me2 marks would be involved in the process.

The non-CG methylation was found to be involved in the epigenetic modification of *OsZIP1* under Cd stress. This is different from the previous observation from Arabidopsis, where non-CG methylation in gene bodies could be hardly detected [38]. It may be the fact that crop plants with larger genomes have much more transposons close to genes and thereby DNA methylation has more responsibilities for gene regulation in crops than in Arabidopsis [38]. DNA methylation at the CG context is found dominantly in the transcribed regions of many constitutively expressed genes [73]. In plants, maintenance of the CG methylation requires MET1 activity [68]. Mutation of *OsMET1* led to a global loss of 75% CmG in rice, causing developmental abnormities [64]. The level of *OsZIP1* methylation in *osmet1* was increased, whereas the *OsZIP1* transcripts were reduced. The mechanism is currently unknown. A recent study has shown that Cd-induced CHG hypomethylation is transgenerationally inherited without entailing an alteration of CG methylation in rice [45]. Gene body methylation is likely established by de novo methylation activity via the RNA-dependent DNA methylation (RdDM) pathway, followed by the maintenance of MET1 [73]. In this case, the occasional antisense transcripts may form the double-stranded RNAs by base-pairing with sense transcripts, leading to generation of small interfering RNAs (siRNAs) which provide a scaffold for DRM2 to methylate the target gene [73]. We examined the *OsZIP1* transcripts in *osdrm2–2* knockout and *osrdr2i-6* knockdown mutant lines but it turned out to have no change in DNA methylation, H3K9me2 marks and transcripts of OsZIP1, indicating that the siRNAs-guided RdDM pathway was not involved in the process.

In animals and human cells, Cd as a non-essential metal is the major cause of many chronic diseases through epigenetic modification such as DNA methylation [75–77]. Maintaining DNA methylation depends on the persistent activity of epigenetic modifiers and disturbing the activity of DNA methyltransferases alters the existing DNA methylation patterns [68]. Cd is a selective inhibitor or stimulator of DNA methyltransferases [75, 76]. The Cd-induced genomic demethylation by inhibiting *DNMT1* and *DNMT3* led to dysregulation of many functional genes [77]. The present study shows that *OsMET1*, *OsCMT3* and *OsSDG714* were transcriptionally downregulated under Cd stress. Although growing evidence shows that Cd is able to change DNA methylation in eukaryotes [37, 78, 79], the direction and specificity of Cd influence on the epigenetic modifications remain to be investigated [77, 79]. The Cd-induced *OsZIP1* expression can be also attributed to the upregulation of some putative transcription factors in the promoter region, as our results show that the *pOsZIP1-GUS* fusion transcripts increased under Cd stress. This is consistent with the study using a DNA methyltransferase inhibitor (5-aza-2-deoxycytidine, Aza) [37], in which *OsZIP1* methylation was reduced with Cd or Aza or Cd + Aza treatment, and its relative expression was significantly increased with +Cd or Aza + Cd samples. The Cd-induced transcripts of OsZIP1 were much more than those of the Aza-treated sample. In this case, Cd not only triggers the promoters but induces demethylation of OsZIP1 as well.

## Conclusions

Based on the results of this study and others, we propose a likely model illustrating the role of *OsZIP1* in transport of its substrates in different ways. Under the normal condition, the low and constant expression of *OsZIP1* in the ER would be required to keep OsZIP1 at a minimum level that ensures proper allocation of Zn and Cu inside cells (or tissues) and prevents the loss of the essential metals. When excess metals are present in the environment, the expression of OsZIP1 can be upregulated. This work will broaden our understanding of the regulatory mechanism underlying cadmium-induced epigenetic modification and metal resistance in rice when challenged to the metal-contaminated soils.

## Methods

### Plant culture and treatment

Rice (*Oryza sativa L. japonica*, c.v. Nipponbare, Kitaake (KT) and Dong Jing (DJ) were used in this study. The T-DNA insertion mutants *Osmet1*, *Oszip1*, *Osdrm2–2*, *Ossdg714* and *Osros1* with their corresponding wild-types were ordered from Kyung Hee University, Korea [51]. Osrdr2–6 RNAi (RNA interference) was kindly provided by Dr. Yi Jun Qi from Tsinhua University, China. Seeds were surface-sterilized by 5% NaClO, rinsed thoroughly with distilled water and germinated under the conditions of 28 °C and darkness for 2 d. After germination, seedlings were transferred to a polyetheylene container, floating on a 0.5 mM CaCl_2_ solution, and grown under the condition of a 14/10 light/dark cycle at 28/25 ± 1 °C (day/night) and 200 μmol m^− 2^ s^− 1^ light intensity for 5 d. The young plants were then transferred to the half-strength Kimura B solution [27]. Plants were then exposed to Cd for a short-term at different concentrations (0–160 μM) based on the previous report [52]. For long-term experiments, rice plants were exposed to 0 and 1 μM Cd for 30 d based on the previous report [27]. Treatment solutions were renewed other day.

### DNA methylation analysis

The sequences of twenty cloned PCR products derived from bisulfite-treated genomic DNA samples were obtained from Feng et al. 2016 [37]. Using these data, the percentages of CG, CHG and CHH methylation were re-analyzed. We then used the McrBC-based DNA methylation assay [80] to validate the results of the bisulfite PCR analysis.

### RNA isolation and transcript analyses by PCR

Total RNA was isolated by TRIzol reagent (Invitrogen) and one μg of total RNA (treated with DNase) was used for quantitative r*everse transcription polymerase chain reaction* (qRT-PCR). One-step gDNA Removal and cDNA Synthesis SuperMix (TransGen Biotech) and qPCR were performed by special primers (Additional file 10: Table S1). *OsACTIN* was used as an internal control with the primers of *OsACTIN-F* (10 μM) and *OsACTIN-R* (10 μM). The samples were pre-incubated at 95 °C for 10 min, followed by 40 cycles of denaturation at 95 °C for 10 s and annealing at 60 °C for 1 min. The reaction was performed in the 7500 real-time PCR System (Applied Biosystems, Foster City, CA, USA) using iTaqTM Universal SYBR Green Supermix (Bio-Rad, Hercules, CA, USA).

### *OsZIP1* transformation in rice

The coding sequence (CDS) of *OsZIP1* (LOC_Os01g74110) was PCR-amplified using the specific primers (Additional file 10: Table S1) [81]. The synthesized *OsZIP1* sequence was digested and inserted into the corresponding sites of *pCAMBIA1300* driven by CaMV35S as a promoter. The embryonic callus of rice induced by mature embryo was infected by *Agrobacterium tumefaciens* carrying *OsZIP1*. For RNAi transformation, a 355 bp cDNA fragment of *OsZIP1* was used and PCR-amplified using the primers listed in Table S1. The PCR products were inserted into the corresponding sites of *LH-FAD1390RNAi*. The constructed vectors were introduced into *A. tumefaciens* EHA105 by thermal activation. At least twenty *35S::OsZIP1* and twenty RNAi lines (T3 homozygotes) were generated.

### Immunoblot detection of *OsZIP1* proteins in plants

Preparing antibody and detecting of *OsZIP1* protein were performed by the method described previously with some modifications [82]. Briefly, polyclonal antibodies were prepared by immunizing Rabbit with purified *OsZIP1* protein. Detection of heat shock protein was used as a control for equal loading. Plant tissues were homogenized in a medium containing 20 mM HEPES (Hepes free acid)-KOH (pH 7.5), 200 mM sorbitol, 1 mM DL-Dithiothreitol, 1 mM Phenylmethanesulfonyl fluoride and a protease inhibitor cocktail. *OsZIP1*-specific antibody was used to detect *OsZIP1* protein.

### Metal quantification

Samples were dried at 70 °C in an air-forced oven for 72 h and weighted. The dried samples were digested with nitric acid. The metal concentrations in the samples were quantified using inductively coupled plasma-atomic emission spectrometry (ICP-AES) (Optimal 2100DV, Perkin Elmer Instruments, Waltham MA, USA) [81].

### Subcellular localization of OsZIP1

The coding sequence of OsZIP1 was amplified by RT-PCR and inserted into pCAMBIA 1305-GFP vector driven by 35S promoter. The OsZIP1-GFP fusion vector was co-transformed with the ER-marker and PM-marker into rice leaf mesophyll protoplasts and tobacco leaves [50]. The fluorescence signal was visualized using Confocal laser scanning microscopy (Confocal System-UitraView VOX, Perkin Elmer).

### Transient expression of *pZIP1::GUS* in tobacco leaves

The 2.1 kb sequence in the *OsZIP1* promoter region was retrieved and constructed into a vector containing GUS (β-glucuronidase) reporter gene. The constructed *pZIP1::GUS* vector was transformed into tobacco (*Nicotiana benthamiana*) leaves by *Agrobacterium tumefaciens* transformation [83]. The transformed cells were exposed to 80 μM Cd for 4 h. qRT-PCR analysis was performed to assess the GUS mRNAs based on the methods described previously [84, 85].

### Chromatin immunoprecipitation assay

The ChIP assay was performed by the method described previously [80]. Anti-H3K9me2 (Abcam; ab1220) antibodies (7 mL) were used. The amount of immunoprecipitated OsZIP1 chromatin was determined by qPCR on different regions of *OsZIP1*. The rice Ubi-10 and Actin1 were used as internal controls. The relative abundance was normalized to the DNA immunoprecipitated by the histone 3-specific antibody.

### Yeast complementation assay

The cDNA fragments containing an entire open reading frame of *OsZIP1* were amplified. The fragments were cloned into pEGM-T Easy vector (with Kpn1 and EcoR1), correctly introduced into vector *pYES2* and incubated at certain condition [49]. The resulting plasmids were transformed into the yeast strain *zrc1* (wild-type BY4741). *zrc1* complementation by drop-spotting assays was performed on the synthetic defined (SD)-Ura medium, which contained 2% galactose, 0.67% yeast nitrogen base (sigma), 2% agar, and supplemented with 0.025, 3 and 6 mM Zn at pH 5.8.

### Statistical analysis

The result was shown as the mean of at least three replicated treatments and each treatment contained at least 9–18 plants. The significant differences between treatments were statistically evaluated by standard deviation (SD) and analysis of variance (ANOVA). The data between differently treated groups were compared statistically by ANOVA followed by the least significant difference (LSD) test if the ANOVA result is significant at *p* < 0.05. The statistic one-way analysis was performed using Statistical Package for the Social Science (SPSS) 22.0 (https://www.ibm.com/analytics/spss-statistics-software).

## Additional files

Additional file 1: **Figure S1.** Basic information of *OsZIP1. (DOC 4190 kb)*

Additional file 2: **Figure S2.** Analysis of OsZIP1 transcripts under normal growth condition. (DOC 794 kb)

Additional file 3: **Figure S3.** Transient expression of GUS reporter genes fused to *OsZIP1* promoter under –Cd and + Cd exposure. (DOC 61 kb)

Additional file 4: **Figure S4.** Identification of *oszip1* mutant, RNAi and OX lines. (DOC 1477 kb)

Additional file 5: **Figure S5.** Zn transport activity and detoxification response assay of of *OsZIP1-*transgenic yeast (*Saccharomyces cerevisiae*). (DOC 1815 kb)

Additional file 6: **Figure S6.** Identification of DNA demethylation of *OsZIP1* in rice exposed to the low level of Cd stress. (DOC 84 kb)

Additional file 7: **Figure S7.** Transcriptional expression of *OsZIP1* under the low level of Cd stress. (DOC 118 kb)

Additional file 8: **Figure S8.** Effects of Cd on the transcripts of DNA methylation modifier genes. (DOC 652 kb)

Additional file 9: **Figure S9.** Effects of Zn, Cu, Mn and Fe excess on the transcription of DNA methylation and demethylation modifier genes. (DOC 3871 kb)

Additional file 10: **Table S1.** Primer sequences used for qRT-PCR in this study. (DOC 42 kb)

## Data Availability

All the data supporting our findings is contained within the manuscript. Constructs and seeds are available upon request from ZMY.

## Abbreviations

Aza: azacitidine
CDS: Coding sequence
ChIP: Chromatin immunoprecipitation
CMT3: CHROMOMETHYLASE3
DJ: Dong Jing
DME: DEMETER
DMGs: Differentially methylated genes
DML2: DEMETER-LIKE2
DRM2: DOMAINS REARRANGED METHYLTRANSFERASE 2)
ER: Endoplasmic reticulum
GFP: Green fluorescent protein
KT: Kitaake
LSD: Least significant difference
MET1: METHYLTRANSFERASE 1
OXs: Overexpressing transgenic lines
PM: Plasma membrane
RNAi: RNA interference
ROS1: REPRESSOR OF SILENCING
TMDs: Transmembrane domains
ZIP: *Z*n-Regulated transporter, *I*ron-regulated transporter-like *P*rotein
