# Supplementary material for: OsZIP1 functions as a metal efflux transporter limiting excess zinc, copper and cadmium accumulation in rice
Source: BMC Plant Biol. 2019 Jun 27;19:283. doi: 10.1186/s12870-019-1899-3 (PMC6598308; doi:10.1186/s12870-019-1899-3)
Supplement: Supplementary file 1 — Figure S1. Basic information of OsZIP1. (DOC 4190 kb) [file 12870_2019_1899_MOESM1_ESM.doc]

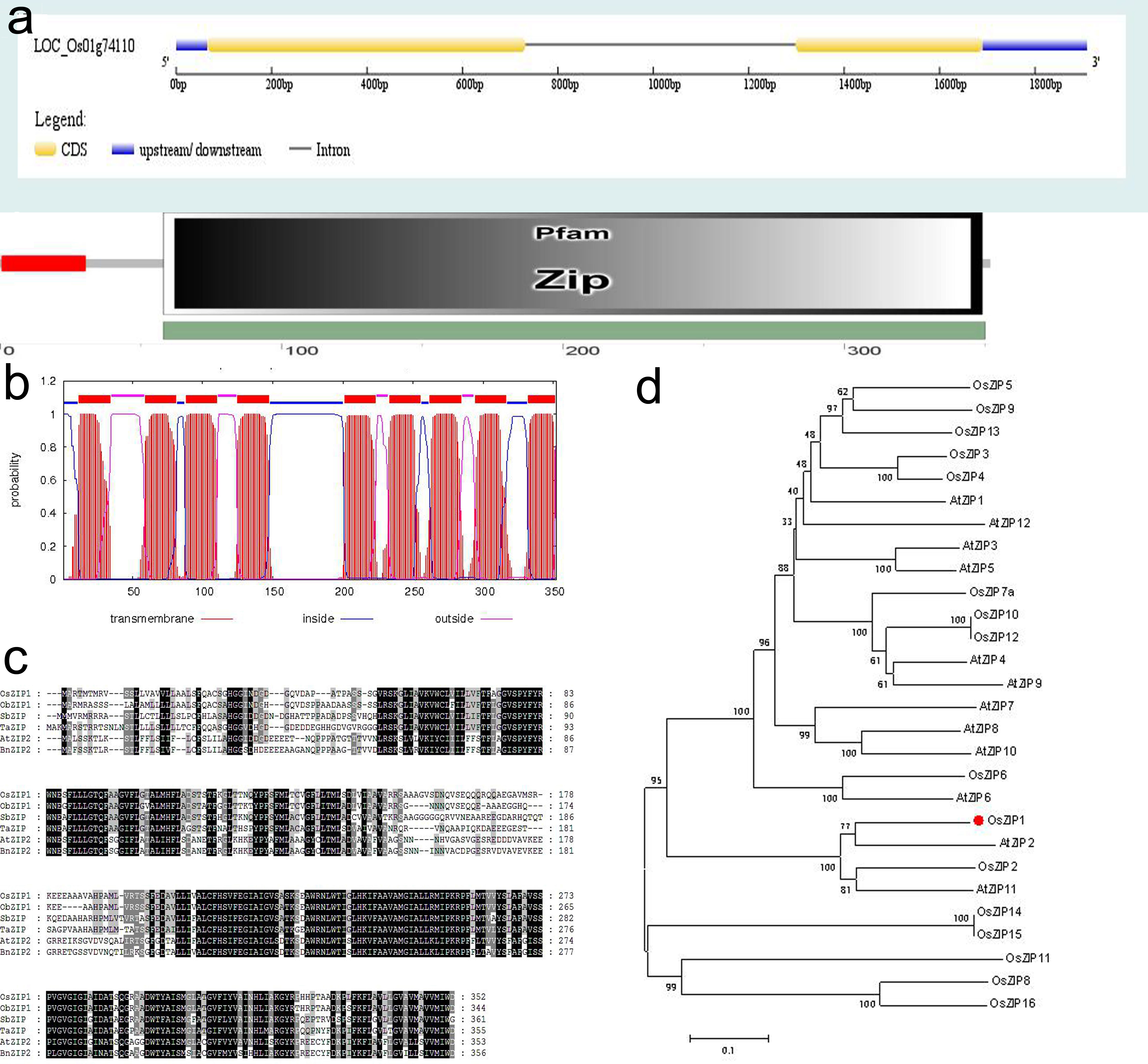


**Additional files 1: Fig. S1** Basic information of *OsZIP1.* **a**: Diagram of the OsZIP1 gene structure. **b**: Transmembrane domain of *OsZIP1* predicted by Simple Modular Architecture Research Tool (SMART, [http://smart.embl-heidelberg.de](http://smart.embl-heidelberg.de/)). **c**: Comparison protein sequences between OsZIP1 and its homologues from different species. **d**: Phylogenetic analysis of OsZIP1 homologous in rice and Arabidopsis.
