# Supplementary material for: OsZIP1 functions as a metal efflux transporter limiting excess zinc, copper and cadmium accumulation in rice
Source: BMC Plant Biol. 2019 Jun 27;19:283. doi: 10.1186/s12870-019-1899-3 (PMC6598308; doi:10.1186/s12870-019-1899-3)
Supplement: Supplementary file 2 — Figure S2. Analysis of OsZIP1 transcripts under normal growth condition. (DOC 794 kb) [file 12870_2019_1899_MOESM2_ESM.doc]

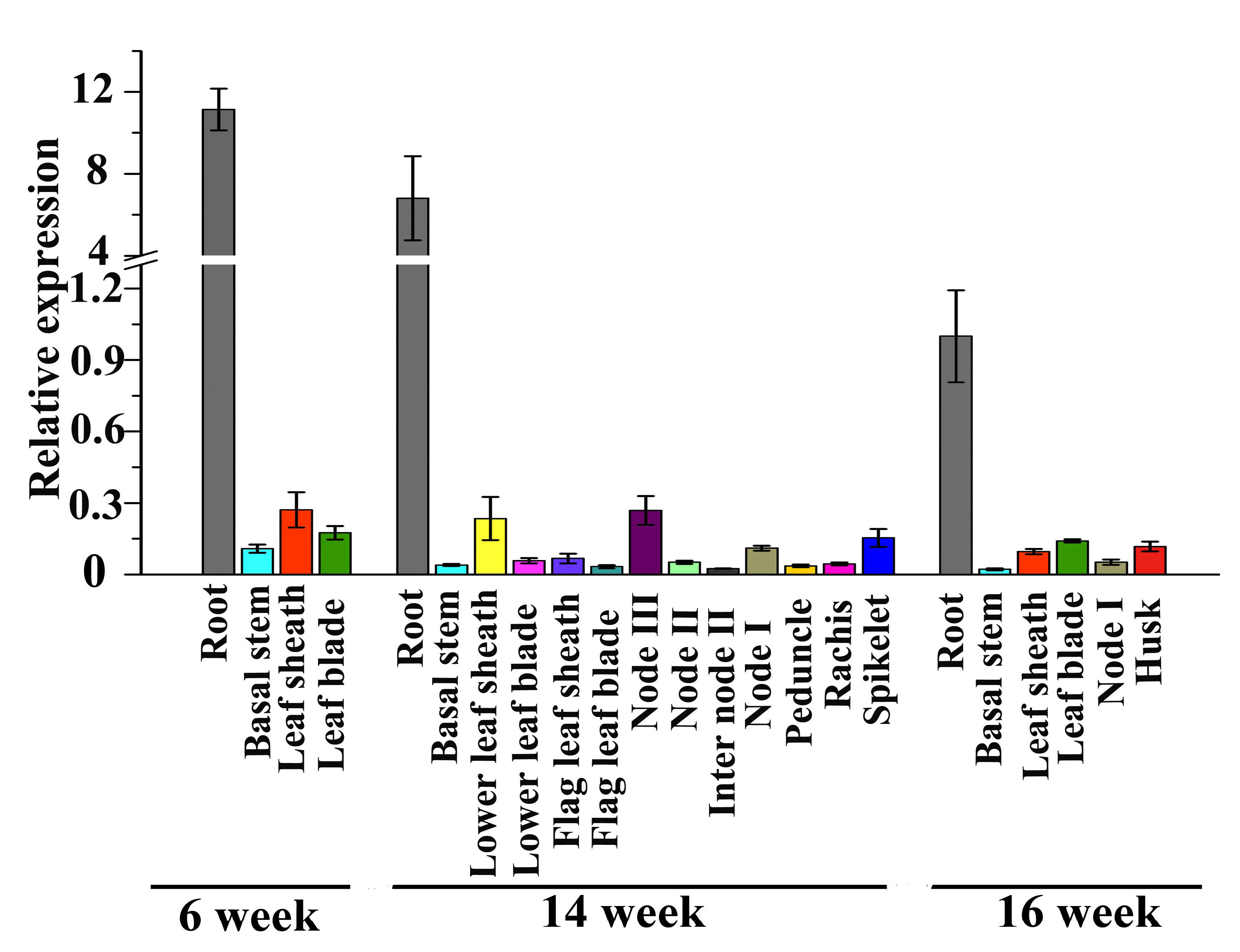


**Additional files 2: Fig. S2**. Analysis of OsZIP1 transcripts under normal growth condition. Germinating young rice plants grew in the nutrient solution and harvested at three developmental stages indicated at 6, 14 and 16 weeks. Total RNA from the samples was isolated and transcripts were determined using qRT-PCR. Vertical bars represent standard deviation.
