# Supplementary material for: OsZIP1 functions as a metal efflux transporter limiting excess zinc, copper and cadmium accumulation in rice
Source: BMC Plant Biol. 2019 Jun 27;19:283. doi: 10.1186/s12870-019-1899-3 (PMC6598308; doi:10.1186/s12870-019-1899-3)
Supplement: Supplementary file 3 — Figure S3. Transient expression of GUS reporter genes fused to OsZIP1 promoter under –Cd and + Cd exposure. (DOC 61 kb) [file 12870_2019_1899_MOESM3_ESM.doc]

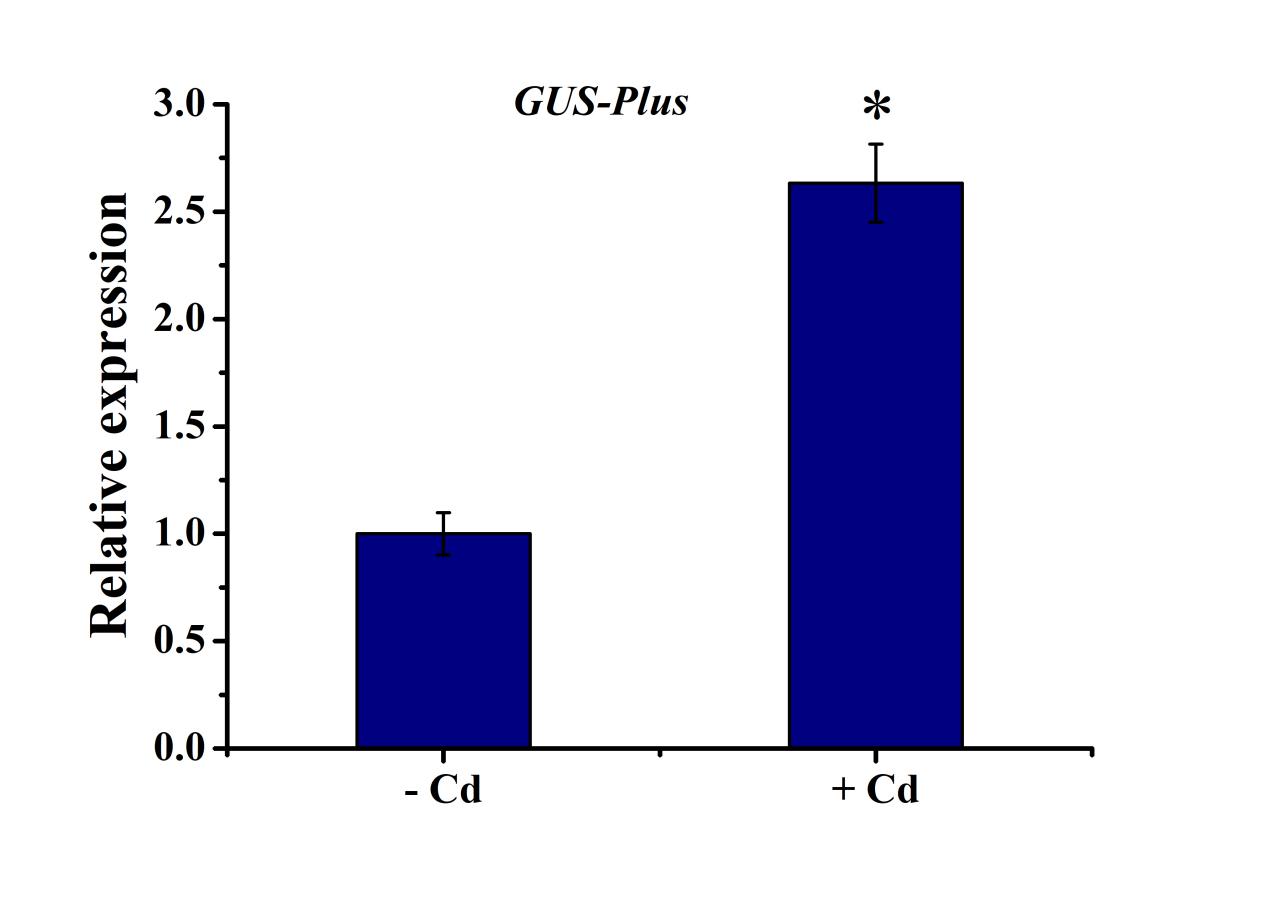


**Additional files 3: Fig. S3**. Transient expression of GUS reporter genes fused to *OsZIP1* promoter under –Cd and +Cd exposure. The transformants carrying *pΔOsZIP1*:GUS vector were exposed to 80 μM Cd for 4 h and qRT-PCR analysis was performed to assess the GUS transcripts. The average GUS expression was obtained from at least five independent transformants and each assay was repeated in triplicate. Vertical bars represent standard deviation. Asterisks indicate that the mean values of three replicates are significantly different between the treatments and control (*p*<0.05).
