# Supplementary material for: OsZIP1 functions as a metal efflux transporter limiting excess zinc, copper and cadmium accumulation in rice
Source: BMC Plant Biol. 2019 Jun 27;19:283. doi: 10.1186/s12870-019-1899-3 (PMC6598308; doi:10.1186/s12870-019-1899-3)
Supplement: Supplementary file 4 — Figure S4. Identification of oszip1 mutant, RNAi and OX lines. (DOC 1477 kb) [file 12870_2019_1899_MOESM4_ESM.doc]

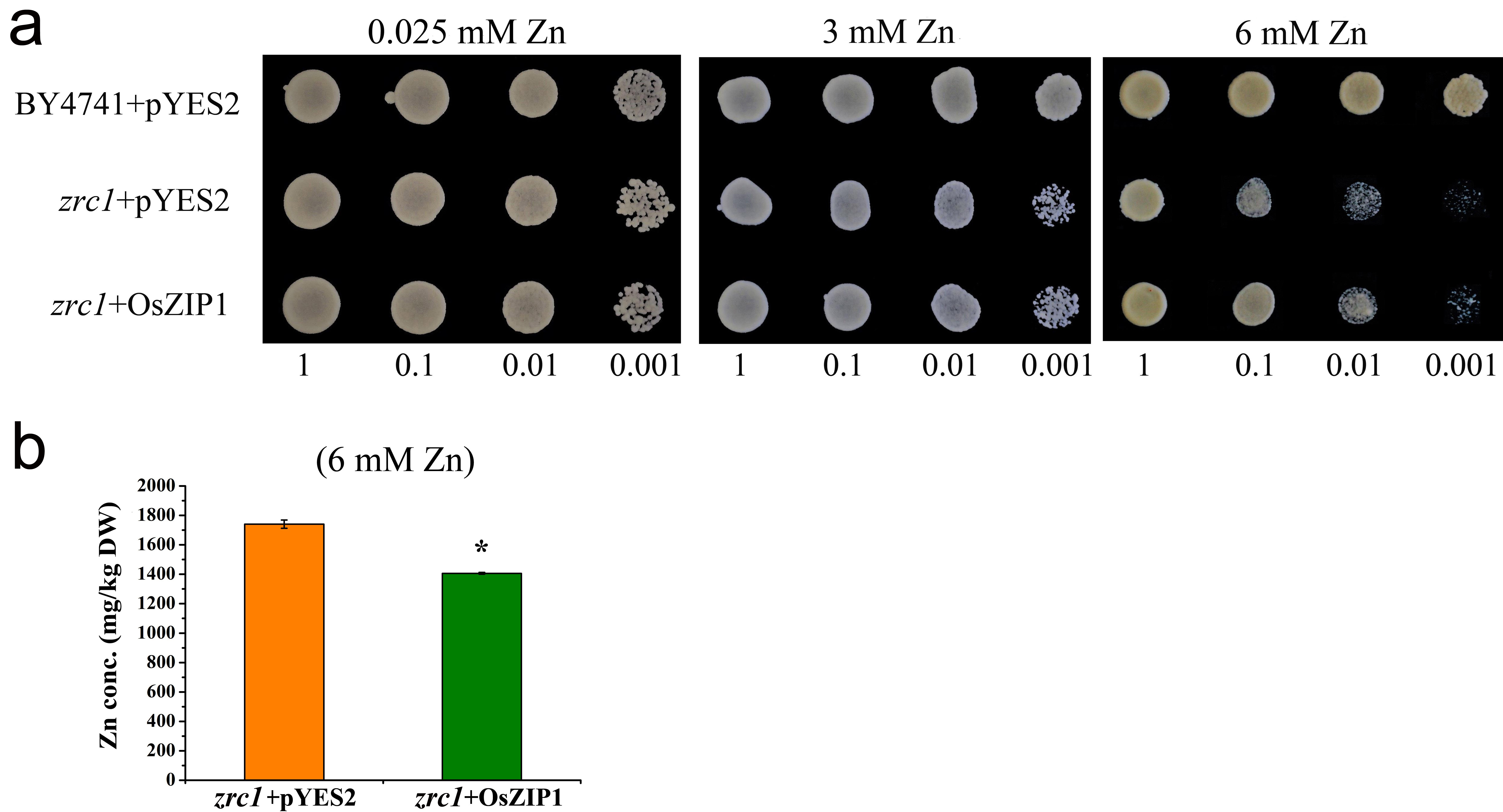


**Additional files 4: Fig. S4**. Zn transport activity and detoxification response assay of of *OsZIP1-*transgenic yeast (*Saccharomyces cerevisiae*). a: Growth of wild-type (BY4741) and *zrc1* mutant cells transformed with pYES2 empty vectors (control) or with pYES2 vectors containing the full *OsZIP1* cDNA were grown in YNB medium supplemented with 0.025 (control), 3 and 6 mM Zn for 3 days. b: Concentrations of Zn in the *OsZIP1* transgenic and empty-vector cells. Vertical bars represent standard deviation of three replicates (20 clones). Asterisks indicate that the mean values are significantly different between the *OsZIP1* transformed cells and empty-vector control (*p*< 0.05).
