# Supplementary material for: OsZIP1 functions as a metal efflux transporter limiting excess zinc, copper and cadmium accumulation in rice
Source: BMC Plant Biol. 2019 Jun 27;19:283. doi: 10.1186/s12870-019-1899-3 (PMC6598308; doi:10.1186/s12870-019-1899-3)
Supplement: Supplementary file 5 — Figure S5. Zn transport activity and detoxification response assay of of OsZIP1-transgenic yeast (Saccharomyces cerevisiae). (DOC 1815 kb) [file 12870_2019_1899_MOESM5_ESM.doc]

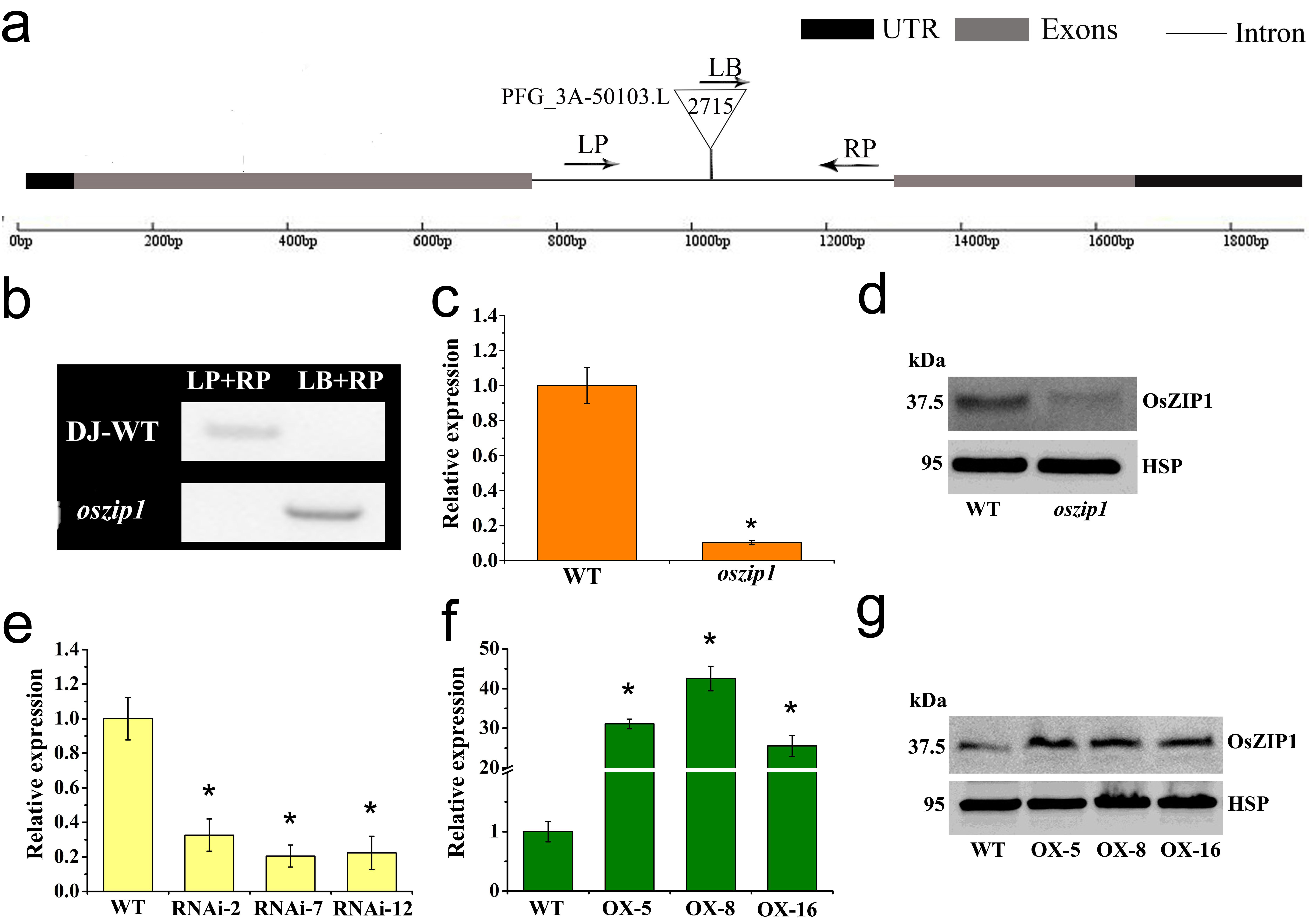


**Additional files 5: Fig. S5**. Identification of *oszip1* mutant, RNAi and OX lines. **a**: Schematic diagram of DNA structures of the mutant locus with a T-DNA insertion into the exon of *OsZIP1.* The untranslated regions are illustrated by the black box. Exons are indicated by grey lines and intron as a line. **b**: identification of mutant homozygote using PCR. **c**: qRT-PCR analysis of the transcriptional expression of *OsZIP1* in the mutant. **d**: identification of the OsZIP1 expression by Western blot. **e**: qRT-PCR analysis of transcriptional expression of *OsZIP1* in RNAi lines. **f**: qRT-PCR analysis of transcriptional expression of *OsZIP1* in *OsZIP1* overexpressing lines (OX). **g**: identification of the OsZIP1 expression in OX by Western blot. Vertical bars represent standard deviation. Asterisks indicate that the mean values of three replicates are significantly different between the wild-type and mutants, RNAi lines or *OsZIP1* overexpressing lines (*p*<0.05).
