# Supplementary material for: OsZIP1 functions as a metal efflux transporter limiting excess zinc, copper and cadmium accumulation in rice
Source: BMC Plant Biol. 2019 Jun 27;19:283. doi: 10.1186/s12870-019-1899-3 (PMC6598308; doi:10.1186/s12870-019-1899-3)
Supplement: Supplementary file 6 — Figure S6. Identification of DNA demethylation of OsZIP1 in rice exposed to the low level of Cd stress. (DOC 84 kb) [file 12870_2019_1899_MOESM6_ESM.doc]

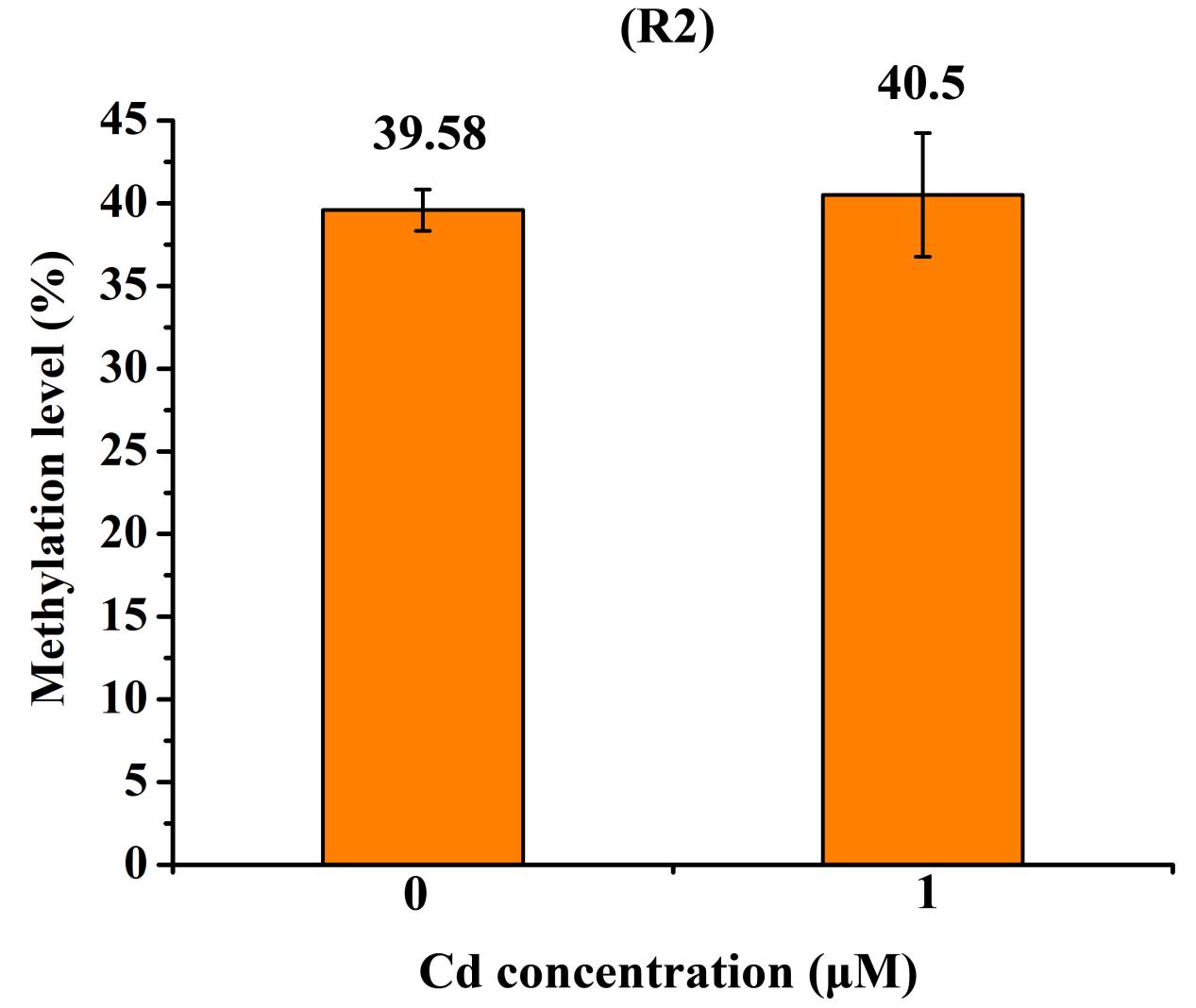


**Additional files 6: Fig. S6**. Identification of DNA demethylation of *OsZIP1* in rice exposed to the low level of Cd stress. Two week-old young rice plants were grown in the nutrient solution supplemented with 0 and 1 µM Cd for 30 d. Total DNA of rice was extracted. BS-PCR analyses were conducted. Vertical bars represent standard deviation.
