# Supplementary material for: OsZIP1 functions as a metal efflux transporter limiting excess zinc, copper and cadmium accumulation in rice
Source: BMC Plant Biol. 2019 Jun 27;19:283. doi: 10.1186/s12870-019-1899-3 (PMC6598308; doi:10.1186/s12870-019-1899-3)
Supplement: Supplementary file 7 — Figure S7. Transcriptional expression of OsZIP1 under the low level of Cd stress. (DOC 118 kb) [file 12870_2019_1899_MOESM7_ESM.doc]

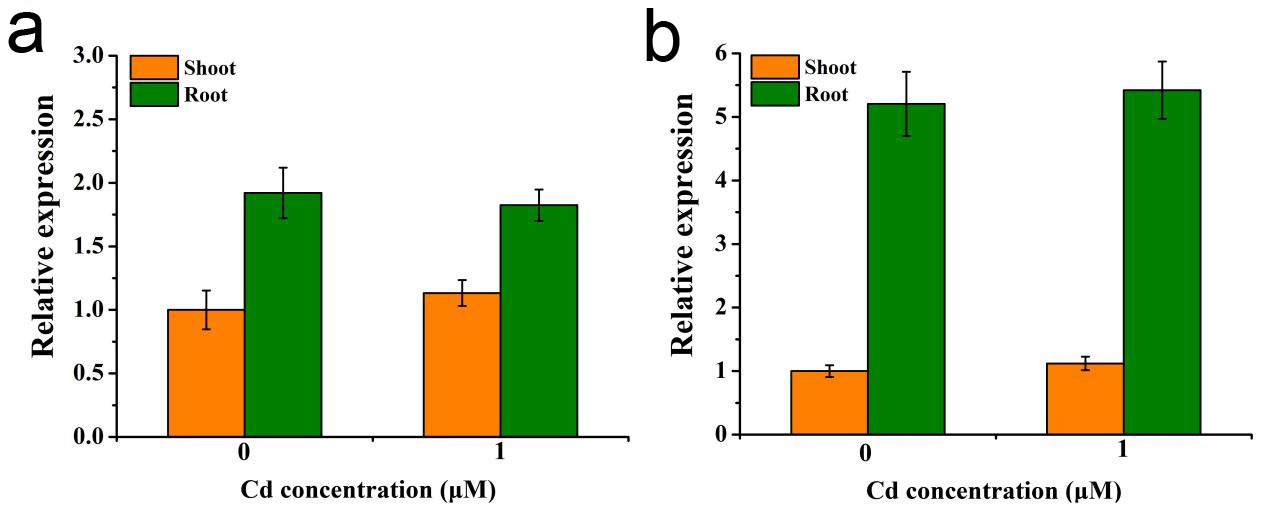


**Additional files 7: Fig. S7**. Transcriptional expression of *OsZIP1* under the low level of Cd stress. **a**: qRT-PCR analysis of gene expression in two week-old rice exposed to Cd at 0 and 1 μM Cd for 3 h. **b**: qRT-PCR analysis of gene expression in two week-old rice exposed to Cd at 0 and 1 μM Cd for 30 d. Vertical bars represent standard deviation (SD) of the mean of three biological replicates.
