# Supplementary material for: OsZIP1 functions as a metal efflux transporter limiting excess zinc, copper and cadmium accumulation in rice
Source: BMC Plant Biol. 2019 Jun 27;19:283. doi: 10.1186/s12870-019-1899-3 (PMC6598308; doi:10.1186/s12870-019-1899-3)
Supplement: Supplementary file 8 — Figure S8. Effects of Cd on the transcripts of DNA methylation modifier genes. (DOC 652 kb) [file 12870_2019_1899_MOESM8_ESM.doc]

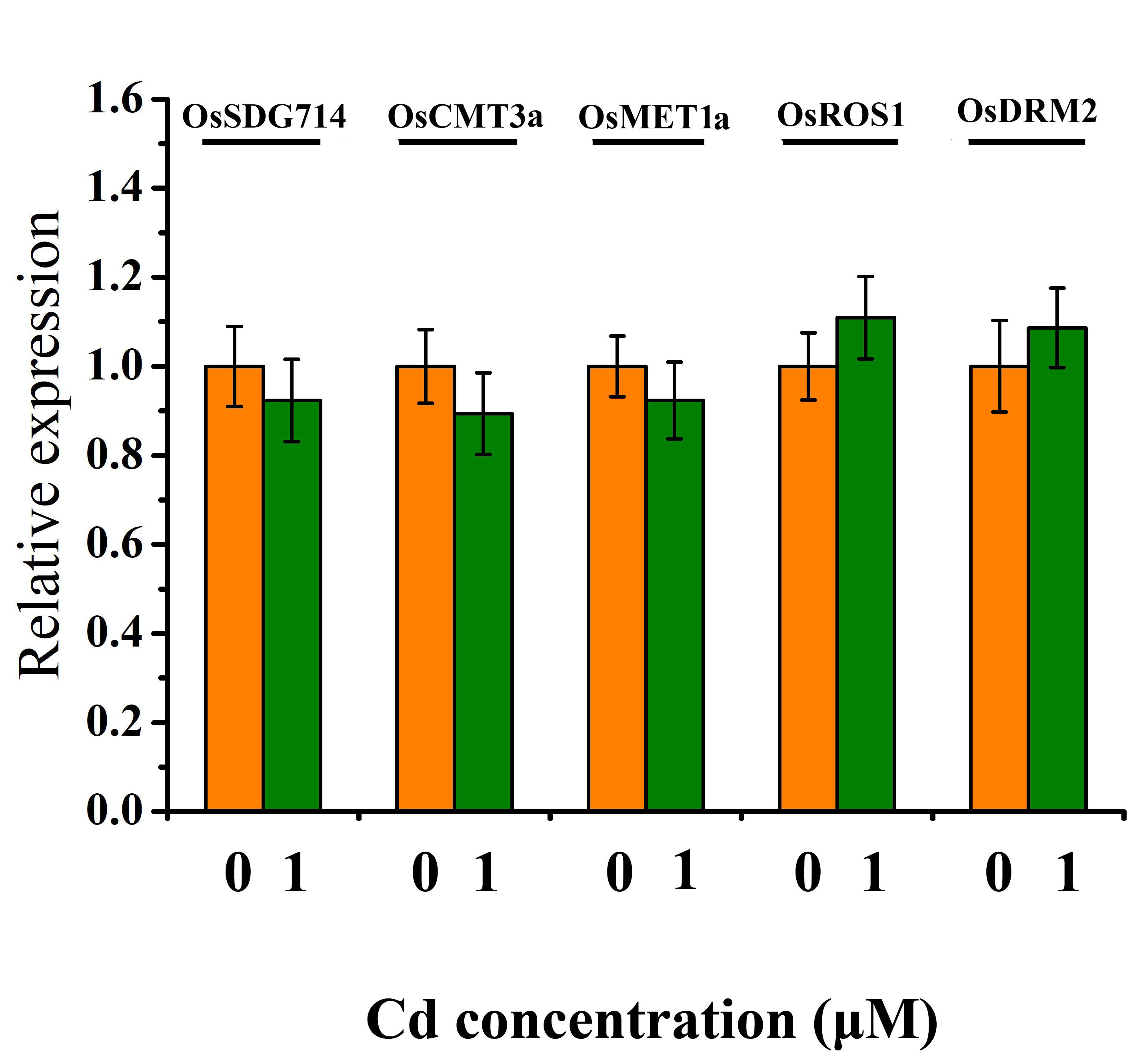


**Additional files 8: Fig. S8**. Effects of Cd on the transcripts of DNA methylation modifier genes. Two week-old young rice plants were grown in the nutrient solution supplemented with 0 and 1 µM Cd for 30 d. Vertical bars represent standard deviation. Asterisks indicate that the mean values of three replicates are significantly different between the treatment and control (*p*<0.05).
