# Supplementary material for: OsZIP1 functions as a metal efflux transporter limiting excess zinc, copper and cadmium accumulation in rice
Source: BMC Plant Biol. 2019 Jun 27;19:283. doi: 10.1186/s12870-019-1899-3 (PMC6598308; doi:10.1186/s12870-019-1899-3)
Supplement: Supplementary file 9 — Figure S9. Effects of Zn, Cu, Mn and Fe excess on the transcription of DNA methylation and demethylation modifier genes. (DOC 3871 kb) [file 12870_2019_1899_MOESM9_ESM.doc]

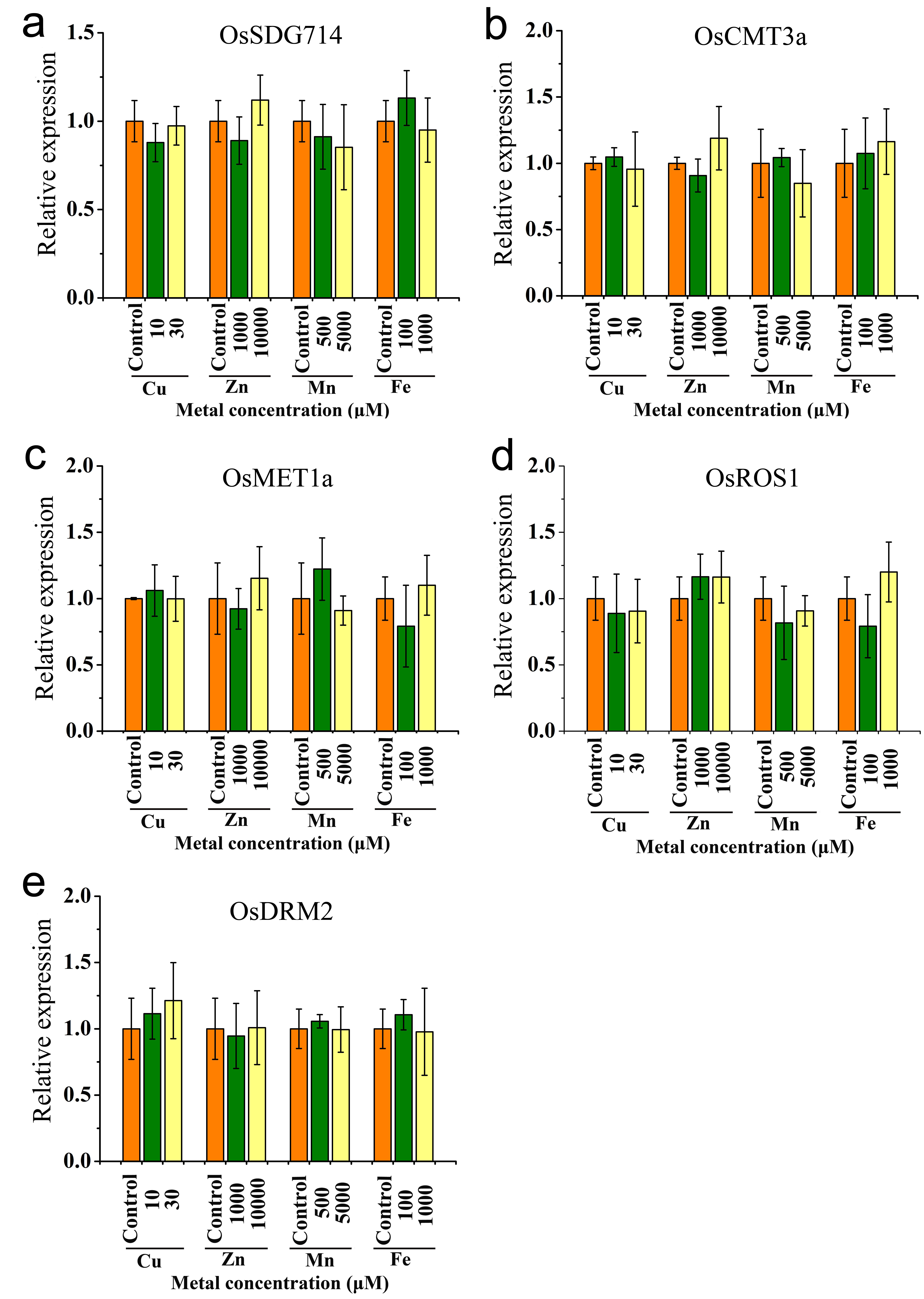


**Additional files 9: Fig. S9**. Effects of Zn, Cu, Mn and Fe excess on the transcription of DNA methylation and demethylation modifier genes. Two week-old young rice plants were grown in the nutrient solution supplemented with the indicated concentrations of the metals for 4 d. Vertical bars represent standard deviation.
