# Supplementary material for: OsZIP1 functions as a metal efflux transporter limiting excess zinc, copper and cadmium accumulation in rice
Source: BMC Plant Biol. 2019 Jun 27;19:283. doi: 10.1186/s12870-019-1899-3 (PMC6598308; doi:10.1186/s12870-019-1899-3)
Supplement: Supplementary file 10 — Table S1. Primer sequences used for qRT-PCR in this study. (DOC 42 kb) [file 12870_2019_1899_MOESM10_ESM.doc]

**Additional files 10: Table S1**. Primer sequences used for qRT-PCR in this study.

|  | Primer name | Forward (5'-3') | Reverse (5'-3') |
| --- | --- | --- | --- |
| qRT-RCR | OsZIP1 | GTGGAGGAACCTGTGGACGA | AGACGACGGTCATGAGGAAG |
|  | OsSDG714 | ACCTGCTGCCGAGGATGATGT | GCCTTCCTGTAACACCGCTTG |
|  | OsMET1a | TAGAGGCAGGGGCTTATGGT | TTGACAGCGGCGTAGAACTT |
|  | OsROS1 | TACTTCCATAGAGACGCAGAC | TCCAGCACTACTTCCAACTACT |
|  | OsCMT3a | AAGCACGACTTGGAATGATGG | ACCACCACGGACAACAACA |
|  | OsDRM2 | AAGTTCGAGTGGGACACAGACG | TTGGCCTTCCCATTTGCATCCTG |
|  | OsActin | GAGTATGATGAGTCGGGTCCAG | ACACCAACAATCCCAAACAGAG |
|  | OsUbiquitin | CGCAAGTACAACCAGGACAA | TGGTTGCTGTGACCACACTT |
|  | OsHistone H3 | GGTCAACTTGTTGATTCCCCTCT | AACCGCAAAATCCAAAGAACG |
|  | OsActin1 | GCCATTCGCCTCCTTCTTG | CCTACTTCAGCAACTCAGTTC |
|  | OsUbi10 | TCACCAGGCTCAGGAAGGA | AGCAGCAGCAAGCATCAAC |
|  | R1 | GAGGAGACTAGAGTCCATATA | TCCTAATTTCAACTATTTCTA |
|  | R2 | AGCTCTCACAATCCAGCCTC | GTCCTGACCTGAGACACCAAT |
|  | R3 | CGTCATGATCTGGGACTGATC | TAACAACTTTTATTCACGGTT |
| Primers used for iPCR of mutants | LB | ACGTCCGCAATGTGTTATTAAG |  |
|  | RB | CAGTCTGGATCGCGAAAACTGTGG |  |
|  | OsZIP1-LP-RP | CTCATCGTCGCGCTCTGTTT | CTCGCTGCAATTCATCTCA |
| Primers used for qPCR of RNAi | OsZIP1 | GACTGGACCTACGCCATCTC | GAACTTGAAGAGCGGCTTGTC |
| Primers used for pCAMBIA 1300 | OsZIP1 | GGGGTACCATGGCCAGGACGATG | CGGGATCCTCAGTCCCAGATCAT |
| Primers used for LH-FAD1390RNAi | OsZIP1 | GGGGTACCCAGCAAGCCGAGGG | CGAGCTCAGGTGGCGTCGATGG |
|  | OsZIP1 | CGGGATCCCAGCAAGCCGAGGG | AACTGCAGAGGTGGCGTCGATGG |
| Primers used for pYES2 | OsZIP1 | GGGGTACCATGGCCAGGACGATG | CGGGATCCTCAGTCCCAGATCAT |
| Primers used for pCAMBIA 1305 | OsZIP1 | GGACTAGTATGGCCAGGACGATG | CGGGATCCGTCCCAGATCAT |
